# Uncertainty Propagation for Inertial Navigation with Coning, Sculling, and Scrolling Corrections

**DOI:** 10.3390/s21248457

**Published:** 2021-12-18

**Authors:** James D. Brouk, Kyle J. DeMars

**Affiliations:** Department of Aerospace Engineering, Texas A&M University, College Station, TX 77843, USA; james.brouk@tamu.edu

**Keywords:** estimation, navigation, uncertainty propagation, coning, sculling, scrolling

## Abstract

This paper investigates the propagation of estimation errors through a common coning, sculling, and scrolling architecture used in modern-day inertial navigation systems. Coning, sculling, and scrolling corrections often have an unaccounted for effect on the error statistics of inertial measurements used to describe the state and uncertainty propagation for position, velocity, and attitude estimates. Through the development of an error analysis for a set of coning, sculling, and scrolling algorithms, mappings of the measurement and estimation errors through the correction term are adaptively generated. Using the developed mappings, an efficient and consistent propagation of the state and uncertainty, within the multiplicative extended Kalman filter architecture, is achieved. Monte Carlo analysis is performed, and results show that the developed system has favorable attributes when compared to the traditional mechanization.

## 1. Introduction

Inertial navigation describes the technique whereby the integration of non-gravitational specific force and total angular rate measurements are used in conjunction with a gravity model to produce estimates for the vehicle states: i.e., the inertial position, velocity, and attitude. In combining the modern inertial navigation system (INS) with the extended Kalman filter (an extension to the Kalman filter [1] for nonlinear systems), estimates for these states and a measure of uncertainty in each can be maintained. Before the introduction of strapdown sensors, inertially stabilized platforms (ISPs) were used to maintain the vehicle’s navigation frame and isolate the necessary sensors from the body’s rotation and any present vibration. A few examples of ISP mechanizations of the INS include the Apollo PGNCS [2], which served on both the command module and lunar module, and those developed for the Minuteman III and Peacekeaper intercontinental ballistic missiles [3]. Though ISPs were the standard until late in the 20th century, they have been widely replaced by the strapdown mechanization, where the sensors are instead “strapped down” or attached directly to the structure of the vehicle [4]. The introduction of strapdown inertial navigation systems (SINSs) significantly reduced the mass and complexity of the INS, as compared to the ISPs, by removing auxiliary components required for the housing and stabilization of ISPs. An early adoption of the SINS for space missions includes the Apollo Abort Guidance System in 1969 as a backup to the PGNCS, where the added mass of a second ISP became a significant consideration [5]. Unfortunately, with the adoption of the SINS, it became necessary to computationally maintain the transformation to the inertially fixed navigation frame as the SINS is not mechanically isolated from the vehicle rotation.

Since the adoption of SINSs, maintaining an accurate attitude estimate has become integral to velocity and position estimation. However, when out-of-phase sinusoidal motion is exhibited about two orthogonal axes, significant error growth is seen [6]—this is commonly referred to as coning motion because, when the angular velocity magnitude and frequency of oscillation are constant, the third axis appears to move on the surface of a cone. These effects are commonly seen in vibrational environments or when maneuvers are performed and are of particular interest to many aerospace applications [7]. A significant portion of SINS development initially focused on generating efficient algorithms capable of addressing the attitude error growth due to coning [8,9,10,11]. The first generation of modern coning correction algorithms was developed by making basic dynamical assumptions and utilizing a sequence of high-frequency inertial measurements [12,13,14], while newer approaches seek to minimize the error under specific circumstances [15,16,17]. Errors can also occur due to sculling and scrolling motions, which are the velocity and position analogs to coning motion, respectively, though they tend not to have the impact that coning does [14]. Fortunately, it has also been shown that once an optimal coning algorithm has been developed, a dual approach for sculling can also be obtained [18,19], extending the usefulness of the approaches. The development of coning, sculling, and scrolling (CSS) algorithms has been critical to SINS-aided navigation, mitigating the effects of cascading attitude errors into the integrated velocity and position. A generalized set of CSS algorithms presented by Savage in [13,14] and their application to modern inertial navigation systems are of primary interest in this paper.

The modern INS is comprised of three orthogonal linear accelerometers and gyroscopes that measure non-gravitational acceleration and total angular velocity, while a navigation computer is employed to integrate these measurements. Because measurements from these sensors help to describe a subset of the vehicle dynamics and are self-contained, they are often called internal or inertial sensors—this helps to differentiate them from other critical measurement systems. Before being passed to the navigation computer, some combination of the CSS corrections is typically applied to the measurements along the processing chain, either within a high-frequency pre-processor or the manufacturer’s sensor software [20,21]. The CSS-corrected measurements are passed to the navigation computer for state propagation. The extended Kalman filter typically serves as the backbone of many modern navigation systems—to accurately propagate the uncertainty for inertial navigation, suitable models for the dynamics and inertial measurements must be known. Unfortunately, inertial measurements are often corrupted by many parametric sensor errors that can have a significant impact on navigation performance [22]. Often, estimates for each of the parametric states are used to correct the inertial measurements before integrating [23]. Thus, the use of CSS-corrected quantities that have been corrected for parametric sensor state estimates contribute to the navigation uncertainty, though this contribution typically goes unaccounted for. By developing and implementing an error mapping through the algorithms used for CSS corrections, a rigorous treatment of these errors and their potential effect on the state uncertainty can be realized.

The remainder of the paper is summarized as follows: Section 2 reviews the mechanics of inertial navigation and presents a standard set of algorithms to make second-order CSS corrections. The primary contribution of the work is contained in Section 3, which outlines the development of the error propagation and presents the mappings for use within the linearized uncertainty propagation. Section 4 describes the approaches to be compared, the lunar descent-to-landing scenario in which the developed methods are examined, and the performance metrics used in analysis. Monte Carlo simulation results are then examined in Section 5. Finally, Section 6 briefly summarizes the work and its primary conclusions.

## 2. Inertial Navigation

**Figure 1:**
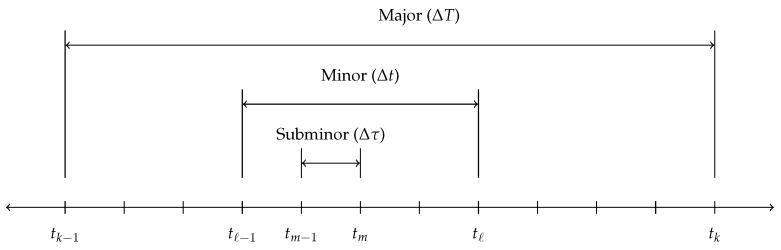
Major, minor, and subminor time intervals considered by the algorithms.

Modern CSS algorithms require several operational frequencies, here defined to be the major, minor, and subminor time intervals illustrated in Figure 1. The subminor interval $\left[t_{m-1},t_{m}\right]$ describes the time over which the inertial sensors integrate to provide discrete measurements of the non-gravitational acceleration and total vehicle angular velocity. The minor interval $\left[t_{\ell -1},t_{\ell }\right]$ defines the rate at which the high-speed correction algorithms are applied, and the major interval $\left[t_{k-1},t_{k}\right]$ defines the navigation rate, or the rate at which the state propagation is performed. For the algorithms considered throughout this paper, the assumption is made that $\left[t_{m-1},t_{m}\right]=\left[t_{\ell -1},t_{\ell }\right]\subseteq \left[t_{k-1},t_{k}\right]$, such that the minor and subminor intervals are equivalent.

The CSS algorithms inspected within this paper generate second-order corrections for coning, sculling, and scrolling motion and are based upon those discussed by Savage in [4,13,14]. These algorithms, along with many other CSS algorithms, were developed after Bortz presented a differential equation of the orientation vector (also called the rotation vector), given as [11]

(1) $$\begin{array}{c} \dot{\phi }=\omega +\frac{1}{2}\phi \times \omega +\frac{1}{\phi^{2}}\left(1-\frac{\phi \sin\phi }{2(1-\cos\phi )}\right)\phi \times \phi \times \omega \;, \end{array}$$

where ϕ is the orientation vector describing the rotation of one frame to another through an angle, ϕ=∥ϕ∥, about an axis pointing in the direction of ϕ, and ω is the angular velocity of the body that is inertially measurable by strapdown angular-rate sensors. Equation (1) is commonly referred to as the Bortz equation and allows for the integration of the orientation vector using measurements from strapdown sensors. Many of the coning algorithms originate from the isolation and approximation of the non-commutative rate vector, $\dot{\alpha }$, in the Bortz equation,

(2) $$\begin{array}{c} \dot{\alpha }=\frac{1}{2}\phi \times \omega +\frac{1}{\phi^{2}}\left(1-\frac{\phi \sin\phi }{2(1-\cos\phi )}\right)\phi \times \phi \times \omega \;. \end{array}$$

A common approximation for Equation (2) is obtained from a power series expansion for the coefficient of the second term in Equation (2), such that

(3) $$\begin{array}{c} \frac{1}{\phi^{2}}\left(1-\frac{\phi \sin\phi }{2(1-\cos\phi )}\right)=\frac{1}{12}\left(1+\frac{1}{60}\phi^{2}+\cdots \right)\approx \frac{1}{12}\;, \end{array}$$

which allows Equation (1) to be approximated as

(4) $$\begin{array}{c} \dot{\phi }\approx \omega +\frac{1}{2}\phi \times \omega +\frac{1}{12}\phi \times \left(\phi \times \omega \right)\;. \end{array}$$

The use of Equation (4) as an approximation for Equation (1) to compensate for coning motion in a two-stage algorithm, which generates the correction at a higher frequency than the state propagation, by Bortz [11] and Jordan [10] laid the foundation for modern coning algorithms. These two-stage approaches generate high-frequency, low-complexity corrections, the results of which are fed into a low-frequency algorithm that produces a state estimate. One of the original two-stage algorithms proposed by Savage in 1966 utilizes a first-order equation at a higher frequency to recognize high-frequency vibrations and a second-order attitude update at a lower frequency, providing an efficient and accurate attitude estimate based upon the corrections made by the high-frequency algorithm [8]. While the two-stage approach was originally introduced because of limited computer capabilities, modern computing capabilities have prompted the desire to return to a single cycle algorithm [13]. However, the algorithms described for most of the two-stage algorithms can also be expanded instead to process a batch of sequential measurements to produce an equivalent, coned measurement at a lower frequency.

The algorithms considered within this paper represent a generalized form of the corrections, assuming that the quantities vary linearly over the minor interval. Additionally, the algorithms examined here are made using only two measurements such that the subminor and minor intervals are the same. Many modern coning correction algorithms have been optimized for error minimization, dependent upon the expected environment or intended number of measurements [12,15,24,25]. It is also important to realize that sculling and scrolling algorithm design has seen much less research and development, leading to a significantly smaller body of literature examining their use. By performing an analysis of the error propagation through unoptimized algorithms, the foundation for analyzing and developing an error propagation architecture for other CSS algorithms used for inertial navigation is established. A detailed derivation of the algorithms is provided by Savage in [4,13,14] and will not be provided within this paper.

### 2.1. Attitude Integration (Coning) Algorithm

The coning algorithm generates a second-order approximation for the coning motion, where the incremental angle vector produced by the inertial measurement unit (IMU) gyroscopes is

(5) $$\begin{array}{c} \Delta \theta_{m}^{c}=\int_{t_{m-1}}^{t_{m}}\omega_{c/i}^{c}\left(\tau \right)d\tau \;, \end{array}$$

where $\omega_{c/i}^{c}$ is the angular velocity vector of the IMU case frame (denoted by *c*) with respect to the inertial frame (denoted by *i*). This incremental angle vector describes the rotation of the IMU case frame from $t_{m-1}$ to $t_{m}$, where the superscript *c* denotes the expression of the vector in the IMU case frame. The frame designation will be implied for the coning elements from here on, as the frames will be consistent throughout the remainder of the paper.

Accumulations for the measurements and corrections made at the end of every minor interval, contained within the major interval, are defined as a function of the incremental angle measurements and used to produce the coned rotation vector. The measurement accumulation is given by

(6) $$\begin{array}{cc} \theta_{\ell } & =\theta_{\ell -1}+\Delta \theta_{\ell }=\sum_{i=1}^{\ell }\Delta \theta_{i}\;. \end{array}$$

The accumulation of the coning corrections is expressed similarly to Equation (6) as

(7) $$\begin{array}{cc} \beta_{\ell } & =\beta_{\ell -1}+\Delta \beta_{\ell }=\sum_{i=1}^{\ell }\Delta \beta_{i}\;, \end{array}$$

where $\Delta \beta_{i}$ is the coning correction generated at $t_{i}$ under the assumption of a linearly varying angular velocity; i.e.,

$$\begin{array}{c} \Delta \beta_{i}=\frac{1}{2}\left[\theta_{i-1}+\frac{1}{6}\Delta \theta_{i-1}\right]\times \Delta \theta_{i}\;. \end{array}$$

Given the accumulations in Equations (6) and (7), the coned rotation vector $\Delta \phi_{k}$ is the sum of the two accumulations, or

(8) $$\begin{array}{c} \Delta \phi_{k}=\theta_{\ell }+\beta_{\ell }\;, \end{array}$$

where $\theta_{\ell }$ describes the sensed change in the attitude over the $\left[t_{k-1},t_{k}\right]$ interval and $\beta_{\ell }$ accounts for the non-commutative and unmeasured components due to the changing angular velocity vector. The *k* subscript denotes that this is the coned rotation vector describing the rotation from $t_{k-1}$ to $t_{k}$, while the *ℓ* subscript describes the number of measurements and corrections accumulated within the major interval.

At the initialization of the algorithm for any given major interval, the terms from the previous time-step must be zero ($\Delta \theta_{0}=0$ and $\Delta \beta_{0}=0$ at $t_{0}=t_{k-1}$). Note that any number of measurements can be processed between the attitude predictions, but when $\left[t_{\ell -1},t_{\ell }\right]=\left[t_{k-1},t_{k}\right]$, or just a single measurement is processed for the prediction, the algorithm becomes identical to traditional methods of dead-reckoning [26], where only a single IMU measurement is processed for the attitude propagation at each step; this statement can be proven by recognizing that with the initialization of the accumulation variables to zero, the correction will also be zero. Finally, if the angular velocity vector is constant in direction, there is no coning motion, the cross product will be zero, and the coning correction in each measurement will thus be zero.

### 2.2. Velocity Integration (Sculling) Algorithm

The velocity integration algorithms correct for errors incurred by IMU frame and velocity vector rotations. The algorithm uses the incremental velocity measurement from the IMU, a quantification of the non-gravitational specific forces acting on the vehicle, given by

$$\begin{array}{cc} \Delta v_{m}^{c} & =\int_{t_{m-1}}^{t_{m}}a_{ng}^{c}\left(\tau \right)d\tau \;, \end{array}$$

where $a_{ng}^{c}$ is the non-gravitational specific force experienced by the strapdown IMU. Note that the superscript *c* denotes the expression of the incremental velocity in the case frame of the IMU; this subscript will also be implied for the sculling elements for the remainder of the paper.

Similar to the accumulation of the measurements in Equation (6), the incremental velocity measurements must also be accumulated, such that

(9) $$\begin{array}{c} v_{\ell }=v_{\ell -1}+\Delta v_{\ell }=\sum_{i=1}^{\ell }\Delta v_{i}\;. \end{array}$$

The sculling-corrected incremental velocity, describing the change in velocity due to the non-gravitational specific forces acting on the vehicle from $t_{k-1}$ to $t_{k}$, can then be separated into three components,

(10) $$\begin{array}{c} \Delta v_{ng,k}=v_{\ell }+\Delta v_{scul,\ell }+\Delta v_{rot,\ell }\;, \end{array}$$

where $\Delta v_{scul,\ell }$ is the sculling correction and $\Delta v_{rot,\ell }$ is the compensation for the rotation of the velocity vector. The *k* and *ℓ* subscripts describe the dependency of the sculled non-gravitational change in velocity upon the *ℓ* inertial measurements obtained over the major interval. The sculling correction can be modeled as an accumulation

(11) $$\begin{array}{cc} \Delta v_{scul,\ell } & =\Delta v_{scul,\ell -1}+\delta v_{scul,\ell }=\sum_{i=1}^{\ell }\delta v_{scul,i}\;, \end{array}$$

where the incremental sculling correction is given by

(12) $$\begin{array}{cc} \delta v_{scul,i} & =\frac{1}{2}\left[\left(\theta_{i-1}+\frac{1}{6}\Delta \theta_{i-1}\right)\times \Delta v_{i}+\left(v_{i-1}+\frac{1}{6}\Delta v_{i-1}\right)\times \Delta \theta_{i}\right]\;, \end{array}$$

which results from an integration of the sculling motion over the measurement cycle, assuming a linearly changing angular velocity and specific force [14]. The correction due to the rotation of the velocity vector on the interval is

(13) $$\begin{array}{c} \Delta v_{rot,\ell }=\frac{1}{2}\left(\theta_{\ell }\times v_{\ell }\right)\;, \end{array}$$

where $\theta_{\ell }$ is the accumulation of the incremental angle measurements as discussed in Section 2.1. The non-gravitational incremental velocity is then used for the vehicle’s velocity propagation.

At the initialization of the algorithm, for any given major interval, the terms from the previous time-step must be zero ($v_{0}=0$ and $\Delta v_{scul,0}=0$ at $t_{0}=t_{k-1}$) because no information is available for the correction on the current interval. Similarly to the coning algorithm, this algorithm can be used to process any number of measurements, and when $\left[t_{\ell -1},t_{\ell }\right]=\left[t_{k-1},t_{k}\right]$, or just a single measurement is used for state prediction, the algorithm becomes identical to the traditional method of dead-reckoning at measurement frequency. This is proven similar to the equivalent statement regarding the coning algorithm.

### 2.3. Position Integration (Scrolling) Algorithm

No additional measurement source is used for the position integration algorithm; the integrated specific force is again integrated to provide the position increment, while the scrolling algorithm corrects for the effects of a varying angular rate and specific forces upon the integration. The effects of scrolling can be accounted for in the non-gravitational specific force integration, given by

(14) $$\begin{array}{c} \Delta r_{ng,k}=s_{v,\ell }+\Delta r_{rot,\ell }+\Delta r_{scrl,\ell }\;. \end{array}$$

The accumulation of the integrated incremental velocity is defined to be

(15) $$\begin{array}{cc} s_{v,\ell } & =s_{v,\ell -1}+\Delta s_{v,\ell }=\sum_{i=1}^{\ell }\Delta s_{v,i}\;, \end{array}$$

where

(16) $$\begin{array}{c} \Delta s_{v,i}=v_{i-1}\Delta t_{\ell }+\frac{1}{12}\left(5\Delta v_{i}+\Delta v_{i-1}\right)\Delta t_{\ell } \end{array}$$

and $\Delta t_{\ell }=t_{\ell }-t_{\ell -1}$. Note that the result in Equation (16) is obtained by integrating the specific force, assuming that the angular velocity and specific force vary linearly over the minor interval [4]. The rotational component of the scrolling correction is given by

(17) $$\begin{array}{c} \Delta r_{rot,\ell }=\frac{1}{6}\left(s_{\theta ,\ell }\times v_{\ell }+\theta_{\ell }\times s_{v,\ell }\right)\;, \end{array}$$

with the integrated incremental angle accumulating as

$$\begin{array}{cc} s_{\theta ,\ell } & =s_{\theta ,\ell -1}+\Delta s_{\theta ,\ell }=\sum_{i=1}^{\ell }\Delta s_{\theta ,i}\;, \end{array}$$

where

$$\begin{array}{c} \Delta s_{\theta ,i}=\theta_{i-1}\Delta t_{\ell }+\frac{1}{12}\left(5\Delta \theta_{i}+\Delta \theta_{i-1}\right)\Delta t_{\ell }. \end{array}$$

The scrolling correction can be broken into a component accounting for the effects due to sculling and a component accounting for other higher-order effects. The accumulation of these effects is therefore given by

(18) $$\begin{array}{c} \Delta r_{scrl,i}=\Delta r_{scrl,i-1}+\delta r_{scrl/scul,i}+\delta r_{scrl/other,i}\;, \end{array}$$

where the scrolling correction contributed by sculling is

(19) $$\begin{array}{cc} \delta r_{scrl/scul,i}= & \;\Delta v_{scul,i-1}\Delta t_{\ell }+\frac{1}{2}\left[\theta_{i-1}-\frac{1}{12}\left(\Delta \theta_{i}-\Delta \theta_{i-1}\right)\right]\times \left(\Delta s_{v,i}-v_{i-1}\Delta t_{\ell }\right)\\ \; & +\frac{1}{2}\left[v_{i-1}-\frac{1}{12}\left(\Delta v_{i}-\Delta v_{i-1}\right)\right]\times \left(\Delta s_{\theta ,i}-\theta_{i-1}\Delta t_{\ell }\right) \end{array}$$

and the correction for the other higher-order effects is

(20) $$\begin{array}{cc} \delta r_{scrl/other,i}=\; & \;\frac{1}{6}\left[s_{v,i-1}+\frac{\Delta t_{\ell }}{24}\left(\Delta v_{i}-\Delta v_{i-1}\right)\right]\times \Delta \theta_{i}\\ \; & -\frac{1}{6}\left[s_{\theta ,i-1}+\frac{\Delta t_{\ell }}{24}\left(\Delta \theta_{i}-\Delta \theta_{i-1}\right)\right]\times \Delta v_{i}\\ \; & +\frac{\Delta t_{\ell }}{6}\left[\theta_{i-1}-\frac{1}{6}\left(\Delta \theta_{i}-\Delta \theta_{i-1}\right)\right]\times \left[v_{i-1}-\frac{1}{6}\left(\Delta v_{i}-\Delta v_{i-1}\right)\right]\\ \; & -\frac{\Delta t_{\ell }}{2160}\left(\Delta \theta_{i}-\Delta \theta_{i-1}\right)\times \left(\Delta v_{i}-\Delta v_{i-1}\right)\;. \end{array}$$

Similar to the coning and sculling algorithms, each term from the previous cycle must be initialized to zero ($s_{\theta ,0}=0$, $s_{v,0}=0$, $\theta_{0}=0$, $\Delta \theta_{0}=0$, $v_{0}=0$ and $\Delta v_{i}=0$ at $t_{0}=t_{k-1}$). It can also be shown that the scrolling correction algorithm and the traditional method for position dead-reckoning are equivalent given that no scrolling motion is present.

### 2.4. Discrete Dead-Reckoning Dynamics

The discretized dynamics for a vehicle aided by a strapdown IMU may be expressed as

(21a) $$\begin{array}{cc} r_{c,k}^{i}=\; & \;r_{c,k-1}^{i}+v_{c,k-1}^{i}\Delta t_{k}+T_{c,k-1}^{i}\Delta r_{ng,k}\\ & \;\;+\frac{1}{2}\left(g_{k-1}-\frac{1}{3}G_{k-1}T_{c,k-1}^{i}\left[d_{k-1}^{c}\times \right]\Delta \phi_{k}\right)\Delta t_{k}^{2} \end{array}$$

(21b) $$\begin{array}{cc} v_{c,k}^{i}= & v_{c,k-1}^{i}+T_{c,k-1}^{i}\Delta v_{ng,k}+\left(g_{k-1}-\frac{1}{2}G_{k-1}T_{c,k-1}^{i}\left[d_{k-1}^{c}\times \right]\Delta \phi_{k}\right)\Delta t_{k} \end{array}$$

(21c) $$\begin{array}{c} {\bar{q}}_{i,k}^{c}=\bar{q}\left(\Delta \phi_{k}\right)⊗{\bar{q}}_{i,k-1}^{c}\;, \end{array}$$

describing the propagation of position r, velocity v and attitude $\bar{q}$ from $t_{k-1}$ to $t_{k}$. Note that the superscripts *c* and *i* denote quantities described in the IMU case frame and inertially fixed reference frame, respectively, while the *k* and k−1 subscripts describe the discrete instances in time $t_{k}$ and $t_{k-1}$, respectively, where the value is available to the navigation computer. Consequently, time time between navigation cycles is thus defined to be $\Delta t_{k}=t_{k}-t_{k-1}$. The transformation from the inertial frame to the case frame is described by the right-handed vector-first attitude quaternion ${\bar{q}}_{i}^{c}$ and transformation matrix $T_{i}^{c}$—the transformation from the case to inertial frame is then given by the transpose of the transformation matrix, ${\left(T_{i}^{c}\right)}^{T}=T_{c}^{i}$. The acceleration acting on the vehicle due to gravity is denoted by $g=a_{g}\left(r_{cg}\right)$, and G is the Jacobian of g evaluated at vehicle center of gravity, $r_{cg}$, where $r_{cg}^{i}=r_{c}^{i}+T_{c}^{i}d^{c}$ relates the position of the vehicle center of gravity $r_{cg}$ to the position of the IMU’s case frame $r_{c}$ via their relative position vector d. The quaternion multiplication is denoted by ⊗ and defined such that the order of quaternion multiplication parallels the multiplication of corresponding transformation matrices. The skew-symmetric cross product matrix is defined to be the matrix equivalent of the cross product operation; i.e., a×b=[a×]b where $a,b\in R^{3}$ and

$$\begin{array}{c} \left[a\times \right]=\left[\begin{array}{ccc} 0 & -a_{3} & a_{2}\\ a_{3} & 0 & -a_{1}\\ -a_{2} & a_{1} & 0 \end{array}\right]\;. \end{array}$$

The total rotation of the body $\Delta \phi_{k}$ is given by Equation (8) and the non-gravitational changes in the position $\Delta r_{ng}$ and velocity $\Delta v_{ng}$ are given by Equations (10) and (14). The incorporation of the CSS corrections accounts for the vector rotation between $t_{k-1}$ to $t_{k}$; this is also done in more traditional approaches [22,26], though the integration of a single set of IMU measurements only provides a first-order correction for the vector rotation. Savage [14] similarly posed these dynamics with the CSS corrections, though he instead considered a rotating navigation frame (such as the East–North–Up (ENU) frame) and implicitly assumed that the origin of the IMU case frame and body’s center of gravity were collocated.

### 2.5. Strapdown IMU Model

The discrete inertial measurements used for the standard SINS are a function of the true quantity being measured and several contributing measurement corruption parameters that result from a variety of sources such as manufacturing tolerances, sensor installation, and unit degradation. The measurement model considered for the strapdown IMU is described by

(22a) $$\begin{array}{cc} \Delta \theta_{m,k} & =\left(I_{3\times 3}+\left[s_{g,k}\setminus \right]\right)\left(I_{3\times 3}+\left[m_{g,k}\times \right]+\left[n_{g,k}*\right]\right)\left(\Delta \theta_{k}+b_{g,k}+w_{g,k}\right) \end{array}$$

(22b) $$\begin{array}{cc} \Delta v_{m,k} & =\left(I_{3\times 3}+\left[s_{a,k}\setminus \right]\right)\left(I_{3\times 3}+\left[m_{a,k}\times \right]+\left[n_{a,k}*\right]\right)\left(\Delta v_{k}+b_{a,k}+w_{a,k}\right)\;, \end{array}$$

where $b_{g,k}$, $s_{g,k}$, $n_{g,k}$, $m_{g,k}$, and $w_{g,k}$ are the bias, scale factor, nonorthogonality, misalignment, and zero-mean, time-wise uncorrelated noise in the gyroscope measurement at $t_{k}$, respectively, while $\Delta \theta_{k}$ is the true incremental angle, and $\Delta \theta_{m,k}$ is the measured incremental angle at $t_{k}$. Similarly, $b_{a,k}$, $s_{a,k}$, $n_{a,k}$, $m_{a,k}$, and $w_{a,k}$ are the bias, scale factor, nonorthogonality, misalignment, and zero-mean, time-wise uncorrelated noise in the accelerometer measurement at $t_{k}$, respectively, $\Delta v_{k}$ is the true incremental velocity, and $\Delta v_{m,k}$ is the measured incremental velocity at $t_{k}$. The operators used in Equation (22) are defined such that, for $y\in R^{3}$,

$$\begin{array}{c} \left[y\setminus \right]=\left[\begin{array}{ccc} y_{1} & 0 & 0\\ 0 & y_{2} & 0\\ 0 & 0 & y_{3} \end{array}\right]\;\;\mathrm{and}\;\left[y*\right]=\left[\begin{array}{ccc} 0 & y_{3} & y_{2}\\ y_{3} & 0 & y_{1}\\ y_{2} & y_{1} & 0 \end{array}\right]\;. \end{array}$$

Applying the models given in Equation (22), the true incremental angle and incremental velocity can be obtained from the measured quantities such that

(23a) $$\begin{array}{cc} \Delta \theta_{k} & ={\left(I_{3\times 3}+\left[m_{g,k}\times \right]+\left[n_{g,k}*\right]\right)}^{-1}{\left(I_{3\times 3}+\left[s_{g,k}\setminus \right]\right)}^{-1}\Delta \theta_{m,k}-b_{g,k}-w_{g,k} \end{array}$$

(23b) $$\begin{array}{cc} \Delta v_{k} & ={\left(I_{3\times 3}+\left[m_{a,k}\times \right]+\left[n_{a,k}*\right]\right)}^{-1}{\left(I_{3\times 3}+\left[s_{a,k}\setminus \right]\right)}^{-1}\Delta v_{m,k}-b_{a,k}-w_{a,k}\;. \end{array}$$

It can then be shown, through the application of the matrix inversion lemma [27], that Equation (23) can be approximated by

(24a) $$\begin{array}{cc} \Delta \theta_{k} & =\Delta \theta_{m,k}-\left[\Delta \theta_{m,k}\setminus \right]s_{g,k}+\left[\Delta \theta_{m,k}\times \right]m_{g,k}-\left[\Delta \theta_{m,k}*\right]n_{g,k}-b_{g,k}-w_{g,k} \end{array}$$

(24b) $$\begin{array}{cc} \Delta v_{k} & =\Delta v_{m,k}-\left[\Delta v_{m,k}\setminus \right]s_{a,k}+\left[\Delta v_{m,k}\times \right]m_{a,k}-\left[\Delta v_{m,k}*\right]n_{a,k}-b_{a,k}-w_{a,k}\;. \end{array}$$

Often, statistics surrounding these parametric errors are studied and detailed by sensor manufacturers, though they can also be determined through independent testing and analysis. If the estimated incremental angle and velocity are denoted as $\Delta {\hat{\theta }}_{i}$ and $\Delta {\hat{v}}_{i}$, respectively, then the error between the true and estimated incremental angle and velocity can be defined such that

(25) $$\begin{array}{c} e_{\Delta \theta ,i}≜\Delta \theta_{i}-\Delta {\hat{\theta }}_{i}\;\mathrm{and}\;e_{\Delta v,i}≜\Delta v_{i}-\Delta {\hat{v}}_{i}\;. \end{array}$$

Fusing the available statistics with the expressions in Equations (24) and (25), a model of how errors in the sensor specific parameters propagate into the IMU measurement errors can be developed. Furthermore, expressions detailing the propagation of these parameteric errors into the state estimation error can be developed and used for implementation within the navigation system architecture.

## 3. Error Propagation Development

By performing the CSS corrections, a correction factor is determined for the raw measurements, allowing the results to better represent the true dynamics of the vehicle. However, when considering the propagation of errors through these corrections, it is clear that if the manufacturer-provided performance specifications for the sensor bias, noise, etc., are for the raw measurements at the sensor’s operational frequency, then these statistics will be inconsistent with the output when CSS corrections are introduced. To have an accurate uncertainty representation, the navigation filter’s covariance prediction requires the description of how errors propagate into the estimate to be representative of the system dynamics. In situations where the navigation system relies upon CSS correction algorithms, the CSS corrections aid in the dynamics description and thus have an influence on the error propagation. Therefore, a rigorous expression for the uncertainty in CSS corrections can be determined and implemented within common navigation system architectures by examining the algorithms and mapping the errors through each.

### 3.1. Method for Error Analysis

To determine how the sensor errors propagate through the CSS algorithms contained within Section 2, the transformation of measurement errors through each correction term is examined. To develop the transformation, it is necessary to consider the estimation error in a given variable to be $e=x-\hat{x}$, such that the error, e, is expressed as the difference between the true, x, and estimated, $\hat{x}$, quantities. Additionally, as seen in Equations (8), (10), and (14), the output of each algorithm can be expressed as a function of the measurement accumulation and the correction terms, while the error dynamics for covariance propagation must be expressed as a function of the estimate and the error in each quantity. To aid in this development, the result of each algorithm is broken into smaller components and recombined to develop the full error dynamics for a given correction.

For example, to determine the coned measurement error, it can be recast as functions of the accumulated incremental angle and coning correction errors such that

(26) $$\begin{array}{c} e_{\Delta \phi ,k}=e_{\theta ,\ell }+e_{\beta ,\ell }\;. \end{array}$$

Noting that the coning correction is a function of the measurements, it is then possible to independently express $e_{\theta ,\ell }$ and $e_{\beta ,\ell }$ as functions of the measurement errors $e_{\Delta \theta ,i}\;\forall i\in \left\{1,2,\cdots \ell \right\}$ as

(27) $$\begin{array}{c} e_{\theta ,\ell }=f\left(e_{\Delta \theta ,i}\right)\;\;\mathrm{and}\;\;e_{\beta ,\ell }=g\left(e_{\Delta \theta ,i}\right)\;, \end{array}$$

where f and g are taken to be independent functions that describe the transformation of sensor errors into the accumulated incremental angle and coning correction vectors, respectively. Therefore, after developing the expressions that map the sensor errors into the accumulation and the correction, the error in the coned measurement can be written as a function of the sensor errors.

The same can be done for the sculled and scrolled non-gravitational changes in velocity and position, respectively. The error mapping is developed by first generating the mapping for each term in the correction individually and then combining the components to construct a mapping of the sensor error sources through the algorithm and into the resulting correction. After developing the mapping of the errors through the algorithms, a slight simplification can be made to each by assuming that some of the error sources are approximately constant over a single major interval. Finally, the transformation of the error into the state estimate can be written as a function of each contributing error source.

### 3.2. Parametric Estimation Errors

The uncertain parameters used to account for sensor errors in IMU measurements are typically identified as neglected, considered, or estimated quantities within the navigation filter, pending the results of a consider analysis as originally posed by S.F. Schmidt [28], though now widely used in application and research [29,30,31,32,33]. In the case in which all of the error parameters are estimated directly by the navigation filter, and assuming that Equation (24) holds, the expected values of the incremental angle $E\left\{\Delta \theta_{k}\right\}=\Delta {\hat{\theta }}_{k}$ and the incremental velocity $E\left\{\Delta v_{k}\right\}=\Delta {\hat{v}}_{k}$ are seen to be

(28a) $$\begin{array}{cc} \Delta {\hat{\theta }}_{k} & =\Delta \theta_{m,k}-\left[\Delta \theta_{m,k}\setminus \right]{\hat{s}}_{g,k}+\left[\Delta \theta_{m,k}\times \right]{\hat{m}}_{g,k}-\left[\Delta \theta_{m,k}*\right]{\hat{n}}_{g,k}-{\hat{b}}_{g,k} \end{array}$$

(28b) $$\begin{array}{cc} \Delta {\hat{v}}_{k} & =\Delta v_{m,k}-\left[\Delta v_{m,k}\setminus \right]{\hat{s}}_{a,k}+\left[\Delta v_{m,k}\times \right]{\hat{m}}_{a,k}-\left[\Delta v_{m,k}*\right]{\hat{n}}_{a,k}-{\hat{b}}_{a,k}\;, \end{array}$$

where ${\hat{b}}_{g,k}$, ${\hat{s}}_{g,k}$, ${\hat{n}}_{g,k}$, and ${\hat{m}}_{g,k}$ are the estimated or expected bias, scale factor, nonorthogonality, and misalignment in the gyroscope measurements, respectively, and ${\hat{b}}_{a,k}$, ${\hat{s}}_{a,k}$, ${\hat{n}}_{a,k}$, and ${\hat{m}}_{a,k}$ are the estimated or expected bias, scale factor, nonorthogonality, and misalignment in the accelerometer measurements, respectively. Note that the noise is defined to be zero-mean. By subtracting Equation (28) from Equation (24) and simplifying, the error in the measurements can be expressed as

(29a) $$\begin{array}{cc} e_{\Delta \theta ,k} & =-\left[\Delta \theta_{m,k}\setminus \right]e_{s_{g},k}+\left[\Delta \theta_{m,k}\times \right]e_{m_{g},k}-\left[\Delta \theta_{m,k}*\right]e_{n_{g},k}-e_{b_{g},k}-w_{g,k} \end{array}$$

(29b) $$\begin{array}{cc} e_{\Delta v,k} & =-\left[\Delta v_{m,k}\setminus \right]e_{s_{a},k}+\left[\Delta v_{m,k}\times \right]e_{m_{a},k}-\left[\Delta v_{m,k}*\right]e_{n_{a},k}-e_{b_{a},k}-w_{a,k}\;, \end{array}$$

where the errors in the bias, scale factor, misalignment, and nonorthogonality estimates for the gyroscopes are defined to be

$$\begin{array}{c} e_{b_{g},k}≜b_{g,k}-{\hat{b}}_{g,k}\;,\;\;e_{s_{g},k}≜s_{g,k}-{\hat{s}}_{g,k}\;,\;\;e_{m_{g},k}≜m_{g,k}-{\hat{m}}_{g,k}\;,\;\;\mathrm{and}\;\;e_{n_{g},k}≜n_{g,k}-{\hat{n}}_{g,k}\;, \end{array}$$

respectively, and, similarly, the errors in the bias, scale factor, misalignment, and nonorthogonality estimates for the accelerometers are

$$\begin{array}{c} e_{b_{a},k}≜b_{a,k}-{\hat{b}}_{a,k}\;,\;\;e_{s_{a},k}≜s_{a,k}-{\hat{s}}_{a,k}\;,\;\;e_{m_{a},k}≜m_{a,k}-{\hat{m}}_{a,k}\;,\;\;\mathrm{and}\;\;e_{n_{a},k}≜n_{a,k}-{\hat{n}}_{a,k}\;, \end{array}$$

respectively. Through this model, sensor errors can be attributed to and expressed as a function of parametric estimation errors.

### 3.3. Coning Error Propagation

Consider the error in the incremental angle internal measurements to be expressed as

(30) $$\begin{array}{c} e_{\theta ,\ell }≜\theta_{\ell }-{\hat{\theta }}_{\ell }\;, \end{array}$$

where $\theta_{\ell }$ and ${\hat{\theta }}_{\ell }$ are the true and estimated accumulated rotation vectors for the major interval, respectively. An expression describing the propagation of errors through the coning algorithm presented in Section 2.1 can be shown to be [34].

(31) $$\begin{array}{c} e_{\Delta \phi }=\sum_{i=1}^{\ell }e_{\Delta \theta ,i}+\Xi_{con,i}e_{\Delta \theta ,i}=\sum_{i=1}^{\ell }\left(I_{3\times 3}+\Xi_{con,i}\right)e_{\Delta \theta ,i}\;, \end{array}$$

where $\Xi_{con,i}≜\left[\xi_{con,i}\times \right]$ and

(32) $$\begin{array}{c} \xi_{con,i}=\frac{1}{2}\left[\sum_{j=1}^{i-1}\Delta {\hat{\theta }}_{j}-\sum_{j=i+1}^{\ell }\Delta {\hat{\theta }}_{j}\right]-\frac{1}{12}\left(\Delta {\hat{\theta }}_{i+1}-\Delta {\hat{\theta }}_{i-1}\right)\;. \end{array}$$

With Equation (32), it can also be shown that the error in the *i*^th^ coning correction term is not correlated to the *i*^th^ measurement, but only to those prior to and following its processing. Additionally, if i+1≥ℓ or i−1≤0, then $\Delta {\hat{\theta }}_{i+1}=0$ or $\Delta {\hat{\theta }}_{i-1}=0$, respectively. To generate $\xi_{con,i}$ as stated in Equation (32), the entire array of *ℓ* measurements must be known; fortunately, this can be restated so that the error terms can be accumulated in a navigation preprocessor algorithm, much like the coning algorithm itself.

### 3.4. Sculling Error Propagation

Through the application of a sculling algorithm, a correction for the measured non-gravitational specific force and its integration into the vehicle’s velocity is made using the incremental angle and velocity measurements over the major interval. It is worth noting that the statistics surrounding the sculling corrected incremental velocity will be dependent upon both the incremental angle and incremental velocity, unlike the coning correction. Noting the equation governing the sculling-corrected non-gravitational term in Equation (10), the error can be written as a sum of errors in each of the components; i.e.,

(33) $$\begin{array}{c} e_{\Delta v_{ng,\ell }}=e_{v,\ell }+e_{\Delta v_{scul},\ell }+e_{\Delta v_{rot},\ell }\;, \end{array}$$

where $e_{v,\ell }$ is the error in the incremental angle accumulation, $e_{\Delta v_{scul},\ell }$ is the error in the sculling correction, and $e_{\Delta v_{rot},\ell }$ is the error in correction for the vehicle’s rotation during the measurement accumulation.

The error in the accumulated incremental velocity can be expressed as

(34) $$\begin{array}{c} e_{v,\ell }≜v_{\ell }-{\hat{v}}_{\ell }\;, \end{array}$$

where $v_{\ell }$ and ${\hat{v}}_{\ell }$ are the true and estimated accumulated velocity vector over the major interval, respectively. By the definition of the incremental velocity accumulation in Equation (9) and the definition of the incremental angle accumulation in Equation (6), it is clear from Equation (25) that the error in each accumulated error is expressed as a sum over all minor interval incremental velocity and angle errors such that

(35) $$\begin{array}{c} e_{v,\ell }=\sum_{i=1}^{\ell }e_{\Delta v,i}\;\mathrm{and}\;e_{\theta ,\ell }=\sum_{i=1}^{\ell }e_{\Delta \theta ,i}\;. \end{array}$$

To determine the error in the sculling correction, first recognize that the sculling correction is a sum of the incremental sculling corrections, as shown in Equation (11), where the increments are defined by Equation (12). Therefore, to determine the error in the sculling correction, the error in the increments must first be determined. Define the error in the sculling increment $e_{\delta v_{scul},i}$ such that

(36) $$\begin{array}{c} e_{\delta v_{scul},i}≜\delta v_{scul,i}-\delta {\hat{v}}_{scul,i}\;. \end{array}$$

Applying the definition of the individual measurement errors and the accumulations, the propagation of errors through the sculling correction is approximated to first-order as

(37) $$\begin{array}{cc} e_{\delta v_{scul},i}= & \left({\hat{\theta }}_{i-1}+\frac{1}{6}\Delta {\hat{\theta }}_{i-1}\right)\times e_{\Delta v,i}-\Delta {\hat{\theta }}_{i}\times \left(e_{v,i-1}+\frac{1}{6}e_{\Delta v,i-1}\right)\\ \; & +\left({\hat{v}}_{i-1}+\frac{1}{6}\Delta {\hat{v}}_{i-1}\right)\times e_{\Delta \theta ,i}-\Delta {\hat{v}}_{i}\times \left(e_{\theta ,i-1}+\frac{1}{6}e_{\Delta \theta ,i-1}\right)\;. \end{array}$$

By the definition of the accumulated incremental sculling corrections given in Equation (11), the accumulated error is simply a sum of the incremental errors; i.e.,

(38) $$\begin{array}{c} e_{\Delta v_{scul},\ell }=\sum_{i=1}^{\ell }e_{\delta v_{scul},i}\;. \end{array}$$

Now that an expression for the error in the accumulated sculling correction is known, the explicit mapping of the error in each measurement into the accumulated error is desired. Notice that Equation (37) has two components that parallel the form of the propagation of the incremental angle measurements through the coning correction—the first is the previously defined $\Xi_{con,i}$, crossed with the incremental velocity errors, while the second term is a similar mapping that can be defined such that $\Xi_{scul,i}≜\left[\xi_{scul,i}\times \right]$, where

(39) $$\begin{array}{c} \xi_{scul,i}=\frac{1}{2}\left[\sum_{j=1}^{i-1}\Delta {\hat{v}}_{j}-\sum_{j=i+1}^{\ell }\Delta {\hat{v}}_{j}\right]-\frac{1}{12}\left(\Delta {\hat{v}}_{i+1}-\Delta {\hat{v}}_{i-1}\right)\;, \end{array}$$

and further allows the definition of the mapping of the measurement errors through the incremental sculling correction to be

(40) $$\begin{array}{c} e_{\Delta v_{scul},\ell }=\sum_{i=1}^{\ell }\left(\Xi_{con,i}e_{\Delta v,i}+\Xi_{scul,i}e_{\Delta \theta ,i}\right)\;. \end{array}$$

The direction of the incremental velocity vector must compensate for the vehicle’s rotation during the major interval; this is done via the rotational correction term. To determine the mapping of the measurement errors through the rotational correction term, define the error in the rotational correction as

(41) $$\begin{array}{c} e_{\Delta v_{rot},\ell }≜\Delta v_{rot,\ell }-\Delta {\hat{v}}_{rot,\ell }\;. \end{array}$$

Expanding Equation (41) with the definition of $\Delta v_{rot,\ell }$ in Equation (13) and simplifying, the error in the rotational correction is then

(42) $$\begin{array}{cc} e_{\Delta v_{rot},\ell }\; & =\frac{1}{2}\left[{\hat{\theta }}_{\ell }\times e_{v,\ell }-{\hat{v}}_{\ell }\times e_{\theta ,\ell }\right]\;, \end{array}$$

when higher-order error terms are neglected. Given that the errors in the accumulations are simply a sum of errors in each of the measurements, the error propagation for the rotational correction is

(43) $$\begin{array}{c} e_{\Delta v_{rot},\ell }=\frac{1}{2}\sum_{i=1}^{\ell }\left(\left[{\hat{\theta }}_{\ell }\times \right]e_{\Delta v,i}-\left[{\hat{v}}_{\ell }\times \right]e_{\Delta \theta ,i}\right)\;. \end{array}$$

To produce the error propagation for the sculled, non-gravitational change in velocity, Equation (33) is combined with the definitions for each component defined in Equations (35), (40), and (43). Therefore, the error in the sculling term as a function of the estimated incremental angles and velocities mapped into the errors in each of those terms is

(44) $$\begin{array}{c} e_{\Delta v_{ng},\ell }=\sum_{i=1}^{\ell }\left(I_{3\times 3}+\Xi_{con,i}+\frac{1}{2}\left[{\hat{\theta }}_{\ell }\times \right]\right)e_{\Delta v,i}+\sum_{i=1}^{\ell }\left(\Xi_{scul,i}-\frac{1}{2}\left[{\hat{v}}_{\ell }\times \right]\right)e_{\Delta \theta ,i}\;. \end{array}$$

The errors in each of these measurement sources, however, is a function of well-known and commonly estimated error sources.

### 3.5. Scrolling Error Propagation

Paralleling the results in Section 3.4, a correction to the measured non-gravitational and its integration into position is made using the incremental angle and velocity measurements by the application of the scrolling algorithm. Whereas the coning correction is dependent upon the statistics surrounding the incremental angle measurements, the scrolling corrections are more similar to the sculling corrections as they are also dependent upon the statistics for the incremental velocity. Noting that the equation governing the scrolling-corrected non-gravitational term is Equation (14), the error can be written as a sum of errors in each of the components; i.e.,

(45) $$\begin{array}{c} e_{\Delta r_{ng},k}=e_{s_{v},\ell }+e_{\Delta r_{rot},\ell }+e_{\Delta r_{scrl},\ell }\;, \end{array}$$

where $e_{s_{v},\ell }$ is the error in the incremental angle accumulation, $e_{\Delta r_{scrl},\ell }$ is the error in the scrolling correction, and $e_{\Delta r_{rot},\ell }$ is the error in the correction for the vehicle’s rotation during the measurement accumulation. Each of these terms is examined separately and recombined to produce the desired result.

The first term in Equation (45) describes the error introduced through the integration of the incremental velocity vectors to determine the change in the vehicle’s position, with the increment and accumulation defined in Equations (15) and (16), respectively. The error must then be expressed in terms of the error in the accumulation and increment; the error in the accumulation is simply a sum of the error in each increment, i.e.,

(46) $$\begin{array}{c} e_{s_{v},\ell }=\sum_{i=1}^{\ell }e_{\Delta s_{v},i}\;. \end{array}$$

The error in the increment is then defined to be the difference between the estimated and true increments, which is given by $e_{\Delta s_{v},i}=\Delta s_{v,i}-\Delta {\hat{s}}_{v,i}$. Substituting for the definition of the increment and truth, the error in the increment can be simplified and expressed as

(47) $$\begin{array}{cc} e_{\Delta s_{v},i} & =e_{v,i-1}\Delta t_{\ell }+\frac{1}{12}\left(5e_{\Delta v,i}+e_{\Delta v,i-1}\right)\Delta t_{\ell }\;, \end{array}$$

allowing Equation (46) to be expressed as a sum of the errors in each increment. Note that the $e_{v,i-1}$ term has been deconstructed and expressed as a sum of the increments by applying Equation (35). Expanding for a variable number of steps, the propagation of incremental velocity errors into the accumulated integrated velocity is given by

(48) $$\begin{array}{c} e_{s_{v},\ell }=\Delta t_{\ell }\sum_{i=1}^{\ell }c_{i,\ell }e_{\Delta v,i}\;, \end{array}$$

with the coefficient $c_{m,n}$ defined to be

(49) $$\begin{array}{c} c_{i,j}=\left\{\begin{array}{cc} \frac{1}{2}+n-m & m<n\\ \frac{5}{12} & m=n\;, \end{array}\right. \end{array}$$

where *m* denotes the index of the measurement error term with which the coefficient is associated, and *n* is the number of measurements contained within the $e_{s_{v},\ell }$ term. In most cases, m=ℓ, though this is not always true.

To accurately propagate the vehicle states with the non-gravitational incremental velocity and angle, the rotation of the vectors during the measurement period is accounted for and defined in Equation (17). When considering uncertainty, however, the transformation of errors in each of the measurements to the correction must be determined. Define the error in the rotational component to be

(50) $$\begin{array}{c} e_{\Delta r_{rot,k}}≜\Delta r_{rot,k}-\Delta {\hat{r}}_{rot,k}, \end{array}$$

and the error in the integrated incremental angle $e_{s_{\theta },\ell }$ can be expressed similarly to how the incremental velocity is defined in Equation (48) as

(51) $$\begin{array}{c} e_{s_{\theta },\ell }=\Delta t_{\ell }\sum_{i=1}^{\ell }c_{i,\ell }e_{\Delta \theta_{i}}\;, \end{array}$$

with $c_{i,\ell }$ defined by Equation (49), where m=i and n=ℓ. It can then be shown that the error in the rotational scrolling term can be expressed as a function of the measurement errors as

(52) $$\begin{array}{c} e_{\Delta r_{rot,k}}=\frac{1}{6}\sum_{i=1}^{\ell }\left(\Lambda_{\Delta \theta ,i}e_{\Delta v_{i}}-\Lambda_{\Delta v,i}e_{\Delta \theta_{i}}\right)\;, \end{array}$$

where $\Lambda_{\Delta \theta ,i}≜\left[\lambda \Delta \theta ,i\times \right]$, $\Lambda_{\Delta v,i}≜\left[\lambda \Delta v,i\times \right]$, and

(53a) $$\begin{array}{cc} \lambda \Delta \theta ,i & ={\hat{s}}_{\theta ,k}+c_{i,\ell }\Delta t_{\ell }{\hat{\theta }}_{k} \end{array}$$

(53b) $$\begin{array}{cc} \lambda \Delta v,i & ={\hat{s}}_{v,k}+c_{i,\ell }\Delta t_{\ell }{\hat{v}}_{k}\;. \end{array}$$

With $\Lambda_{\Delta \theta ,i}$ and $\Lambda_{\Delta v,i}$ defined, the mapping of measurement errors through the position rotational correction is known.

The scrolling correction term, as described by Equation (18), is composed of an accumulation of two incremental corrections: a correction for the presence of sculling motion and its integration into the position and a correction for the presence of other, higher-order effects. The error in the accumulation of the scrolling correction can be expressed as

(54) $$\begin{array}{c} e_{\Delta r_{scrl},\ell }=\sum_{i=1}^{\ell }e_{\delta r_{scrl/scul},i}+\sum_{i=1}^{\ell }e_{\delta r_{scrl/other},i}\;, \end{array}$$

where the error in each increment is defined to be

(55a) $$\begin{array}{cc} e_{\delta r_{scrl/scul},i} & ≜\delta r_{scrl/scul,i}-\delta {\hat{r}}_{scrl/scul,i} \end{array}$$

(55b) $$\begin{array}{cc} e_{\delta r_{scrl/other},i} & ≜\delta r_{scrl/other,i}-\delta {\hat{r}}_{scrl/other,i}\;. \end{array}$$

Applying the definition in Equation (19) to Equation (55a), the propagation of sensor errors through the incremental scrolling correction for sculling motion is determined to be

(56) $$\begin{array}{cc} e_{\delta r_{scrl/scul},i} & =\Delta t_{\ell }\sum_{j=1}^{i-1}\left(\Xi_{con,j}e_{\Delta v,j}+\Xi_{scul,j}e_{\Delta \theta ,j}\right)\\ \; & \;-\frac{\Delta t_{\ell }}{24}\left(5\Delta {\hat{\theta }}_{i}+\Delta {\hat{\theta }}_{i-1}\right)\times e_{v,i-1}-\frac{\Delta t_{\ell }}{24}\left(5\Delta {\hat{v}}_{i}+\Delta {\hat{v}}_{i-1}\right)\times e_{\theta ,i-1}\\ \; & \;+\frac{\Delta t_{\ell }}{24}\left({\hat{\theta }}_{i-1}-\frac{1}{2}\Delta {\hat{\theta }}_{i}\right)\times e_{\Delta v,i-1}+\frac{\Delta t_{\ell }}{24}\left({\hat{v}}_{i-1}-\frac{1}{2}\Delta {\hat{v}}_{i}\right)\times e_{\Delta \theta ,i-1}\\ \; & \;+\frac{\Delta t_{\ell }}{24}\left(5{\hat{\theta }}_{i-1}+\frac{1}{2}\Delta {\hat{\theta }}_{i-1}\right)\times e_{\Delta v,i}+\frac{\Delta t_{\ell }}{24}\left(5{\hat{v}}_{i-1}+\frac{1}{2}\Delta {\hat{v}}_{i-1}\right)\times e_{\Delta \theta ,i}\;. \end{array}$$

It is worth noting the dependency of Equation (56) on the sculling correction, as expected. For brevity, the derivation of Equation (56) is neglected here but can be found in [35].

Using the expression describing the propagation of measurement errors through the incremental correction, the propagation of the measurement errors through the scrolling algorithm’s correction for sculling motion is simply a sum of errors in each increment generated across the major interval. Expanding manually, it can be shown that the error in the incremental scrolling correction for sculling motion is then expressed as

(57) $$\begin{array}{cc} e_{\Delta r_{scrl/scul},\ell } & =\Delta t_{\ell }[\sum_{i=1}^{\ell }\left(\left(\ell -i\right)\Xi_{con,i}+\frac{1}{24}\Gamma_{\Delta \theta ,i}\right)e_{\Delta v,i}\\ \; & \;\;+\sum_{i=1}^{\ell }\left(\left(\ell -i\right)\Xi_{scul,i}+\frac{1}{24}\Gamma_{\Delta v,i}\right)e_{\Delta \theta ,i}]\;, \end{array}$$

with the mappings defined such that $\Gamma_{\Delta \theta ,i}≜\left[\gamma_{\Delta \theta ,i}\times \right]$ and $\Gamma_{\Delta v,i}≜\left[\gamma_{\Delta v,i}\times \right]$, where

$$\begin{array}{c} \gamma_{\Delta \theta ,i}=\left\{\begin{array}{cc} 6\left(\sum_{j=1}^{i-1}\Delta {\hat{\theta }}_{j}-\sum_{j=i+1}^{\ell }\Delta {\hat{\theta }}_{j}\right)+\frac{1}{2}\left(\Delta {\hat{\theta }}_{i-1}-\Delta {\hat{\theta }}_{i+1}\right)+\Delta {\hat{\theta }}_{\ell } & i<\ell \\ 5\sum_{j=1}^{i-1}\Delta {\hat{\theta }}_{j}+\frac{1}{2}\Delta {\hat{\theta }}_{i-1} & i=\ell \end{array}\right. \end{array}$$

and

$$\begin{array}{c} \gamma_{\Delta v,i}=\left\{\begin{array}{cc} 6\left(\sum_{j=1}^{i-1}\Delta {\hat{v}}_{j}-\sum_{j=i+1}^{\ell }\Delta {\hat{v}}_{j}\right)+\frac{1}{2}\left(\Delta {\hat{v}}_{i-1}-\Delta {\hat{v}}_{i+1}\right)+\Delta {\hat{v}}_{\ell } & i<\ell \\ 5\sum_{j=1}^{i-1}\Delta {\hat{v}}_{j}+\frac{1}{2}\Delta {\hat{v}}_{i-1} & i=\ell \;. \end{array}\right. \end{array}$$

The scrolling correction error introduced by the additional correction for higher-order effects is a sum of the error in each incremental correction made over the major interval. In any given increment, the propagation of each measurement error into the scrolling correction increment for higher-order effects can be defined such that [35]

(58) $$\begin{array}{cc} e_{\delta r_{scrl/other},i}= & \frac{\Delta t_{\ell }}{6}\sum_{j=1}^{i-1}\left(\left[{\hat{\theta }}_{i-1}-\frac{1}{6}\left(\Delta {\hat{\theta }}_{i}-\Delta {\hat{\theta }}_{i-1}\right)-c_{j,\;i-1}\Delta {\hat{\theta }}_{i}\right]\times e_{\Delta v,j}\right)\\ \; & -\frac{\Delta t_{\ell }}{6}\sum_{j=1}^{i-1}\left(\left[{\hat{v}}_{i-1}-\frac{1}{6}\left(\Delta {\hat{v}}_{i}-\Delta {\hat{v}}_{i-1}\right)-c_{j,\;i-1}\Delta {\hat{v}}_{i}\right]\times e_{\Delta \theta ,j}\right)\\ \; & +\frac{\Delta t_{\ell }}{6}\left[\frac{1}{6}{\hat{\theta }}_{i-1}-\frac{1}{40}\left(\Delta {\hat{\theta }}_{i}-\Delta {\hat{\theta }}_{i-1}\right)+\Delta {\hat{\theta }}_{i}\right]\times e_{\Delta v,i-1}\\ \; & -\frac{\Delta t_{\ell }}{6}\left[\frac{1}{6}{\hat{v}}_{i-1}-\frac{1}{40}\left(\Delta {\hat{v}}_{i}-\Delta {\hat{v}}_{i-1}\right)+\Delta {\hat{v}}_{i}\right]\times e_{\Delta \theta ,i-1}\\ \; & +\frac{\Delta t_{\ell }}{6}\left[\frac{7}{120}\left(\Delta {\hat{\theta }}_{i}-\Delta {\hat{\theta }}_{i-1}\right)-\frac{1}{24}\Delta {\hat{\theta }}_{i-1}+\sum_{j=1}^{i-1}\left(i-j-\frac{1}{3}\right)\Delta {\hat{\theta }}_{j}\right]\times e_{\Delta v,i}\\ \; & -\frac{\Delta t_{\ell }}{6}\left[\frac{7}{120}\left(\Delta {\hat{v}}_{i}-\Delta {\hat{v}}_{i-1}\right)-\frac{1}{24}\Delta {\hat{v}}_{i-1}+\sum_{j=1}^{i-1}\left(i-j-\frac{1}{3}\right)\Delta {\hat{v}}_{j}\right]\times e_{\Delta \theta ,i}\;, \end{array}$$

where the coefficient $c_{j,i-1}$ is defined in Equation (49), with m=j and n=i−1. The propagation of errors into the accumulated correction is then the sum of contributions for a given measurement error into each of the increments. Through a manual expansion of the terms, an expression mapping measurement errors into the scrolling correction term that accounts for higher-order effects is expressed as

(59) $$\begin{array}{c} e_{\Delta r_{scrl/other},\ell }=\sum_{i=1}^{\ell }e_{\delta r_{scrl/other},i}=\frac{\Delta t_{\ell }}{6}\sum_{i=1}^{\ell }\left(M_{\Delta v,i}e_{\Delta \theta ,i}-M_{\Delta \theta ,i}e_{\Delta v,i}\right)\;, \end{array}$$

with $M_{\Delta \theta ,i}≜\left[\mu_{\Delta \theta ,i}\times \right]$ and $M_{\Delta v,i}≜\left[\mu_{\Delta v,i}\times \right]$, where $\mu_{\Delta \theta ,i}$ and $\mu_{\Delta v,i}$ can be expressed as

(60) $$\begin{array}{c} \mu_{\Delta \theta ,i}=\left\{\begin{array}{cc} \left(\ell -i+\frac{1}{6}\right){\hat{\theta }}_{i}-\frac{1}{10}\Delta {\hat{\theta }}_{i-1}+\frac{5}{4}\Delta {\hat{\theta }}_{i}+\frac{13}{120}\Delta {\hat{\theta }}_{i+1}-\frac{1}{6}\Delta {\hat{\theta }}_{\ell } & i<\ell \\ \;\;\;+\sum_{j=1}^{i-1}\left(i-j-\frac{1}{3}\right)\Delta {\hat{\theta }}_{j}+\sum_{j=i+1}^{\ell }\left(\ell +i-2j+\frac{1}{2}\right)\Delta {\hat{\theta }}_{j} & \\ \; & \\ \frac{7}{120}\Delta {\hat{\theta }}_{i}-\frac{1}{10}\Delta {\hat{\theta }}_{i-1}+\sum_{j=1}^{i-1}\left(i-j-\frac{1}{3}\right)\Delta {\hat{\theta }}_{j} & i=\ell \end{array}\right. \end{array}$$

and

(61) $$\begin{array}{c} \mu_{\Delta v,i}=\left\{\begin{array}{cc} \left(\ell -i+\frac{1}{6}\right){\hat{v}}_{i}-\frac{1}{10}\Delta {\hat{v}}_{i-1}+\frac{5}{4}\Delta {\hat{v}}_{i}+\frac{13}{120}\Delta {\hat{v}}_{i+1}-\frac{1}{6}\Delta {\hat{v}}_{\ell } & i<\ell \\ \;\;\;+\sum_{j=1}^{i-1}\left(i-j-\frac{1}{3}\right)\Delta {\hat{v}}_{j}+\sum_{j=i+1}^{\ell }\left(\ell +i-2j+\frac{1}{2}\right)\Delta {\hat{v}}_{j} & \\ \; & \\ \frac{7}{120}\Delta {\hat{v}}_{i}-\frac{1}{10}\Delta {\hat{v}}_{i-1}+\sum_{j=1}^{i-1}\left(i-j-\frac{1}{3}\right)\Delta {\hat{v}}_{j} & i=\ell \;. \end{array}\right. \end{array}$$

The error in the scrolling-corrected non-gravitational change in position can be expressed by substituting the components from Equations (48), (52), (57), and (59), the propagation of errors is expressed as

(62) $$\begin{array}{cc} e_{\Delta r_{ng,k}}= & \sum_{i=1}^{\ell }\left(X_{\Delta v,i}e_{\Delta \theta_{i}}+X_{\Delta \theta ,i}e_{\Delta v_{i}}\right)\;, \end{array}$$

where

$$\begin{array}{cc} X_{\Delta v,i}= & \Delta t_{\ell }\left(\left(\ell -i\right)\Xi_{scul,i}+\frac{1}{24}\Gamma_{\Delta v,i}+\frac{1}{6}M_{\Delta v,i}\right)-\frac{1}{6}\Lambda_{\Delta v,i}\\ X_{\Delta \theta ,i}= & \Delta t_{\ell }\left(c_{i,\ell }I_{3\times 3}+\left(\ell -i\right)\Xi_{con,i}+\frac{1}{24}\Gamma_{\Delta \theta ,i}-\frac{1}{6}M_{\Delta \theta ,i}\right)+\frac{1}{6}\Lambda_{\Delta \theta ,i} \end{array}$$

are defined to simplify notation.

### 3.6. State Estimation Error Dynamics

For implementation within the multiplicative extended Kalman filter (MEKF) [36], a widely used tool developed to appropriately handle the quaternion representation of attitude, the transformation of errors through each of the CSS algorithms must be incorporated into the relationships describing the propagation of state estimation errors, where errors in the position and velocity states are defined to be

$$\begin{array}{c} e_{r,k}≜r_{c,k}^{i}-{\hat{r}}_{c,k}^{i}\;\mathrm{and}\;e_{v,k}≜v_{c,k}^{i}-{\hat{v}}_{c,k}^{i}\;, \end{array}$$

respectively. Unlike position and velocity, the attitude quaternions may not be directly subtracted. Thus, the attitude error is instead defined by the multiplicative attitude error; i.e.,

(63) $$\begin{array}{c} \delta {\bar{q}}_{i,k}^{c}≜{\bar{q}}_{i,k}^{c}⊗{\left({\hat{\bar{q}}}_{i,k}^{c}\right)}^{-1}\;, \end{array}$$

as is necessary according to the MEKF architecture. Under a small angle assumption, the error in the attitude is approximately given by

$$\begin{array}{c} e_{a,k}=2\delta q_{i,k}^{c}\;, \end{array}$$

where $\delta q_{i,k}^{c}$ is the vector part of the error quaternion.

Examining the discrete-time dynamics in Equation (21), it can be shown that the error equations for the position, velocity, and attitude of the vehicle are given by [35]

$$\begin{array}{cc} e_{r,k}= & \left\{I_{3\times 3}+\frac{\Delta t_{k}^{2}}{2}\left({\hat{g}}_{k-1}-\frac{1}{3}{\hat{U}}_{k-1}\right)\right\}e_{r,k-1}+\Delta t_{k}e_{v,k-1}\\ \; & +\{\frac{\Delta t_{k}^{2}}{6}{\hat{g}}_{k-1}{\hat{T}}_{k-1}^{T}\left[\left({\hat{d}}_{k-1}\times \Delta {\hat{\phi }}_{k}\right)\times \right]-{\hat{T}}_{k-1}^{T}\left[\Delta {\hat{r}}_{ng,k}\times \right]\\ \; & \;\;-\frac{\Delta t_{k}^{2}}{2}\left({\hat{g}}_{k-1}-\frac{1}{3}{\hat{U}}_{k-1}\right){\hat{T}}_{k-1}^{T}\left[{\hat{d}}_{k-1}\times \right]\}e_{a,k-1}\\ \; & +\frac{\Delta t_{k}^{2}}{2}\left\{\left({\hat{g}}_{k-1}-\frac{1}{3}{\hat{U}}_{k-1}\right){\hat{T}}_{k-1}^{T}+\frac{1}{3}{\hat{g}}_{k-1}{\hat{T}}_{k-1}^{T}\left[\Delta {\hat{\phi }}_{k}\times \right]\right\}e_{d,k-1}\\ \; & +{\hat{T}}_{k-1}^{T}e_{\Delta r_{ng},k}-\frac{\Delta t_{k}^{2}}{6}{\hat{g}}_{k-1}{\hat{T}}_{k-1}^{T}\left[{\hat{d}}_{k-1}\times \right]e_{\Delta \phi ,k}\\ e_{v,k}= & \Delta t_{k}\left({\hat{g}}_{k-1}-\frac{1}{2}{\hat{U}}_{k-1}\right)e_{r,k-1}+e_{v,k-1}\\ \; & +\{\frac{\Delta t_{k}}{2}{\hat{g}}_{k-1}{\hat{T}}_{k-1}^{T}\left[\left({\hat{d}}_{k-1}\times \Delta {\hat{\phi }}_{k}\right)\times \right]-{\hat{T}}_{k-1}^{T}\left[\Delta {\hat{v}}_{ng,k}\times \right]\\ \; & \;\;-\Delta t_{k}\left({\hat{g}}_{k-1}-\frac{1}{2}{\hat{U}}_{k-1}\right){\hat{T}}_{k-1}^{T}\left[{\hat{d}}_{k-1}\times \right]\}e_{a,k-1}\\ \; & +\Delta t_{k}\left\{\left({\hat{g}}_{k-1}-\frac{1}{2}{\hat{U}}_{k-1}\right){\hat{T}}_{k-1}^{T}+\frac{1}{2}{\hat{g}}_{k-1}{\hat{T}}_{k-1}^{T}\left[\Delta {\hat{\phi }}_{k}\times \right]\right\}e_{d,k-1}\\ \; & +{\hat{T}}_{k-1}^{T}e_{\Delta v_{ng},k}-\frac{\Delta t_{k}}{2}{\hat{g}}_{k-1}{\hat{T}}_{k-1}^{T}\left[{\hat{d}}_{k-1}\times \right]e_{\Delta \phi ,k}\\ e_{a,k}= & T\left(\Delta {\hat{\phi }}_{k}\right)e_{a,k-1}+e_{\Delta \phi ,k} \end{array}$$

This result is similar to that of more traditional methods where only a single set of IMU measurements is used for propagation, as is seen in [26], though the contribution from the incremental angle and velocity are removed and housed within the $e_{\Delta v_{ng},k}$ and $e_{\Delta r_{ng},k}$ terms. Having previously described the transformation of errors through the CSS algorithms in Equations (31), (44), and (62), the state estimation error dynamics can then be expressed as

(64a) $$\begin{array}{cc} e_{r,k} & =\left\{I_{3\times 3}+\frac{\Delta t_{k}^{2}}{2}\left({\hat{g}}_{k-1}-\frac{1}{3}{\hat{U}}_{k-1}\right)\right\}e_{r,k-1}+\Delta t_{k}e_{v,k-1}\\ \; & \;+\{\frac{\Delta t_{k}^{2}}{6}{\hat{g}}_{k-1}{\hat{T}}_{k-1}^{T}\left[\left({\hat{d}}_{k-1}\times \Delta {\hat{\phi }}_{k}\right)\times \right]-{\hat{T}}_{k-1}^{T}\left[\Delta {\hat{r}}_{ng,k}\times \right]\\ \; & \;\;-\frac{\Delta t_{k}^{2}}{2}\left({\hat{g}}_{k-1}-\frac{1}{3}{\hat{U}}_{k-1}\right){\hat{T}}_{k-1}^{T}\left[{\hat{d}}_{k-1}\times \right]\}e_{a,k-1}\\ \; & \;+\frac{\Delta t_{k}^{2}}{2}\left\{\left({\hat{g}}_{k-1}-\frac{1}{3}{\hat{U}}_{k-1}\right){\hat{T}}_{k-1}^{T}+\frac{1}{3}{\hat{g}}_{k-1}{\hat{T}}_{k-1}^{T}\left[\Delta {\hat{\phi }}_{k}\times \right]\right\}e_{r_{cg/c},k-1}\\ \; & \;+\sum_{i=1}^{\ell }\left\{{\hat{T}}_{k-1}^{T}X_{\Delta v,i}-\frac{\Delta t_{k}^{2}}{6}{\hat{g}}_{k-1}{\hat{T}}_{k-1}^{T}\left[{\hat{d}}_{k-1}\times \right]\left(I_{3\times 3}+\Xi_{con,i}\right)\right\}e_{\Delta \theta ,i}\\ \; & \;+\sum_{i=1}^{\ell }{\hat{T}}_{k-1}^{T}X_{\Delta \theta ,i}e_{\Delta v,i} \end{array}$$

(64b) $$\begin{array}{cc} e_{v,k} & =\Delta t_{k}\left({\hat{g}}_{k-1}-\frac{1}{2}{\hat{U}}_{k-1}\right)e_{r,k-1}+e_{v,k-1}\\ \; & \;+\{\frac{\Delta t_{k}}{2}{\hat{g}}_{k-1}{\hat{T}}_{k-1}^{T}\left[\left({\hat{d}}_{k-1}\times \Delta {\hat{\phi }}_{k}\right)\times \right]-{\hat{T}}_{k-1}^{T}\left[\Delta {\hat{v}}_{ng,k}\times \right]\\ \; & \;\;-\Delta t_{k}\left({\hat{g}}_{k-1}-\frac{1}{2}{\hat{U}}_{k-1}\right){\hat{T}}_{k-1}^{T}\left[{\hat{d}}_{k-1}\times \right]\}e_{a,k-1}\\ \; & \;+\Delta t_{k}\left\{\left({\hat{g}}_{k-1}-\frac{1}{2}{\hat{U}}_{k-1}\right){\hat{T}}_{k-1}^{T}+\frac{1}{2}{\hat{g}}_{k-1}{\hat{T}}_{k-1}^{T}\left[\Delta {\hat{\phi }}_{k}\times \right]\right\}e_{d,k-1}\\ \; & \;+\sum_{i=1}^{\ell }\left\{{\hat{T}}_{k-1}^{T}\left(\Xi_{scul,i}-\frac{1}{2}\left[{\hat{v}}_{\ell }\times \right]\right)-\frac{\Delta t_{k}}{2}{\hat{g}}_{k-1}{\hat{T}}_{k-1}^{T}\left[{\hat{d}}_{k-1}\times \right]\left(I_{3\times 3}+\Xi_{con,i}\right)\right\}e_{\Delta \theta ,i}\\ \; & \;+\sum_{i=1}^{\ell }{\hat{T}}_{k-1}^{T}\left(I_{3\times 3}+\Xi_{con,i}+\frac{1}{2}\left[{\hat{\theta }}_{\ell }\times \right]\right)e_{\Delta v,i} \end{array}$$

(64c) $$\begin{array}{l} e_{a,k}=T\left(\Delta {\hat{\phi }}_{k}\right)e_{a,k-1}+e_{\Delta \phi ,k}\;. \end{array}$$

Note that $e_{d,k}$ is the error in the position of the vehicle center of gravity with respect to the IMU case frame origin, and ${\hat{U}}_{k-1}$ arises due to this discrepancy in the vehicle’s center of gravity position and is defined to have an element in the *i*^th^ row and *j*^th^ column given by [26]

$$\begin{array}{c} {\hat{U}}_{k-1}\left(i,j\right)=\left[{\left.\sum_{m=1}^{3}\frac{\partial^{2}g\left(i\right)}{\partial r(j)\partial r(m)}u\left(m\right)\right|}_{r={\hat{r}}_{cg,k-1}}\right]\;, \end{array}$$

where

$$\begin{array}{c} u_{k-1}={\hat{T}}_{k-1}^{T}\left[{\hat{d}}_{k-1}\times \right]\Delta {\hat{\phi }}_{k}\;. \end{array}$$

It should be noted that r(j) and r(i) denote the *i*^th^ and *j*^th^ elements of the $r_{cg,k-1}$ vector, while g(i) similarly denotes the *i*^th^ component of $g_{k-1}$ and u(m) denotes the *m*^th^ component of $u_{k-1}$. Finally, notice that the frame designations have been dropped from the transformation matrix—it is assumed that the transformation matrix T represents the transformation from the inertial frame to the case frame.

### 3.7. Covariance Propagation

Let the navigation filter state vector ${\hat{x}}_{k}$ be given by the concatenation of the estimated position, velocity, and attitude states of the vehicle, augmented by the estimated inertial sensor parameters, or

$$\begin{array}{c} {\hat{x}}_{k}=\left[\begin{array}{c} {\hat{r}}_{c,k}^{i}\\ {\hat{v}}_{c,k}^{i}\\ {\hat{\bar{q}}}_{i,k}^{c}\\ {\hat{p}}_{g,k}\\ {\hat{p}}_{a,k} \end{array}\right]\;,\;{\hat{p}}_{g,k}=\left[\begin{array}{c} {\hat{b}}_{g,k}\\ {\hat{s}}_{g,k}\\ {\hat{m}}_{g,k}\\ {\hat{n}}_{g,k} \end{array}\right]\;,\;\mathrm{and}\;{\hat{p}}_{a,k}=\left[\begin{array}{c} {\hat{b}}_{a,k}\\ {\hat{s}}_{a,k}\\ {\hat{m}}_{a,k}\\ {\hat{n}}_{a,k} \end{array}\right]\;, \end{array}$$

where ${\hat{p}}_{g,k}$ and ${\hat{p}}_{a,k}$ are the concatenated sensor error parameters for the gyroscope and accelerometers, respectively. Notice that the state estimation error is defined to be

$$\begin{array}{c} e_{k}=x_{k}-{\hat{x}}_{k} \end{array}$$

for all states except for the attitude, which requires the multiplicative error definition for the attitude quaternion in Equation (63). Thus, the dynamics governing the estimation error can, as given in Equation (64), can be expressed as

$$\begin{array}{c} e_{k}=F_{k-1}e_{k-1}+M_{k-1}w_{k-1}\;, \end{array}$$

where $F_{k-1}$ is the discrete-time dynamics Jacobian that describes the mapping of errors from one time-step to the next, while $M_{k-1}$ maps dynamic process noise into the state estimation error. It is worth pointing out that, for systems aided by strapdown inertial sensors, the dynamic process noise described by $w_{k-1}$ is taken to be the noise of the inertial sensors; i.e,

$$\begin{array}{c} w_{k-1}=\left[\begin{array}{c} w_{a,k-1}\\ w_{g,k-1} \end{array}\right]\;. \end{array}$$

After combining Equation (29) with Equation (64), the elements of $F_{k-1}$ and $M_{k-1}$ are given by inspection. For brevity, this combination is done in Appendix A. From this definition of the state estimation error dynamics, the linearized covariance propagation can be clearly stated such that

$$\begin{array}{c} P_{k}=F_{k-1}P_{k-1}F_{k-1}^{T}+M_{k-1}q_{k-1}M_{k-1}^{T}\;, \end{array}$$

where $Q_{k-1}$ is the process noise covariance. Note that, for the typical INS, the uncertainty introduced by noise in strapdown sensor measurements is commonly incorporated as process noise.

## 4. Simulation

Two separate architectures are examined for a lunar descent-to-landing simulation. The first architecture utilizes a traditional dead-reckoning architecture—i.e., one propagating after receiving a single incremental angle and velocity [22,26]—while the other employs the CSS algorithms and utilizes the rigorous treatment of the estimation error propagation as developed within Section 3. The same trajectory is examined in [22,37], where the effects of external measurements on the navigation performance are considered. In contrast, the analysis here focuses on the situation where the vehicle is only navigating through inertial navigation techniques and thus has no external measurements to aid in navigation. Understanding how the estimation error propagates through the navigation system in a high-stakes scenario, such as a lunar landing, is crucial for informing future systems’ development. Comparing the two different propagation techniques with this scenario provides a quantifiable differentiation between navigation systems equipped with the CSS algorithms’ error propagation and those without. It should be noted that the selected trajectory is chosen not to maximize the effects or usefulness of CSS algorithms but to represent a scenario in which these algorithms may be used on a realistic trajectory in which increased accuracy and precision is desired. This comparison highlights the expected difference in navigation system performance when employing the error propagation alongside the often-implemented CSS algorithms.

To assess performance and compare configurations, Monte Carlo analysis is used. However, this method of analysis typically samples the true state from some distribution about the mean. Breaking convention, the true position, velocity, attitude, acceleration, and angular velocity are fixed for this trajectory, as is the case in [37], requiring that the initial estimates be sampled from a distribution about the true states. The initial states are assumed to be initially uncorrelated and sampled from Gaussian distributions, with 1σ uncertainties in each channel of position, velocity, and attitude shown in Table 1.

**Figure 2:**
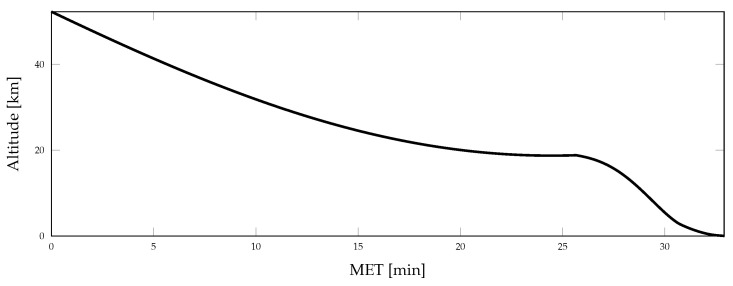
Vehicle altitude during terminal descent.

**Figure 3:**
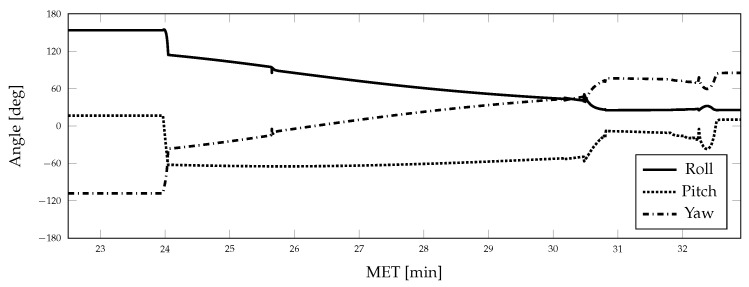
Vehicle attitude (Euler angles) during terminal descent.

**Figure 4:**
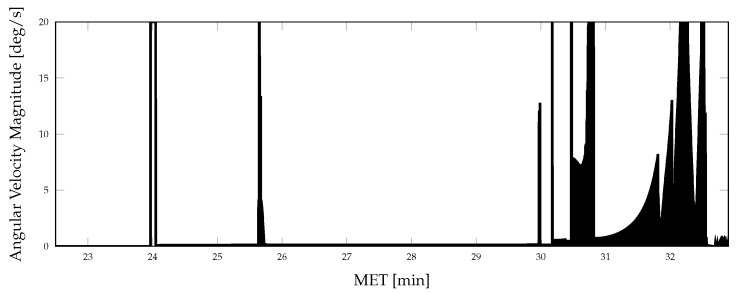
Vehicle angular velocity magnitude during terminal descent.

**Figure 5:**
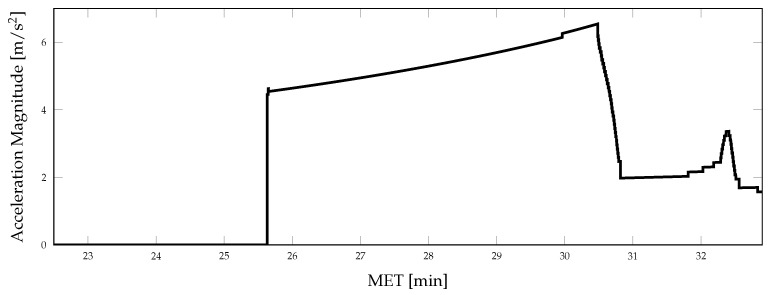
Non-gravitational acceleration magnitude during terminal descent.

Aspects of the true vehicle trajectory are illustrated in Figure 2, Figure 3 and Figure 4. The altitude profile of the vehicle across the mission is shown in Figure 2, where the trajectory is initialized 50 km above the lunar surface. The vehicle slowly descends over the first 24 min to an altitude of 16.5 km and enters a powered descent phase after 25.5 min of mission elapsed time (MET). During powered descent, the vehicle rapidly descends to the surface in just under 7 min. Figure 3 shows the vehicle attitude, expressed as Euler angles; the attitude profile is only provided for a portion of the mission to show the changes experienced throughout the powered descent phase of the simulation. A large attitude maneuver occurs at approximately 24 min MET, as can be seen in Figure 3 and Figure 4, while a smaller attitude maneuver occurs at roughly 25.5 min MET, coinciding with the start of the powered descent phase. During the powered descent phase, the vehicle performs a translational maneuver to decelerate and land the vehicle, as seen in Figure 5. As the simulation ends and the vehicle approaches its landing location, several small attitude correction maneuvers are also performed.

The IMU gyroscope and accelerometer measurements utilize the model given in Section 2.5 and have sensor error statistics consistent with the Northrop Grumman LN-200S [38], as provided in Table 2. Measurement error sources of white noise, bias, scale factor, misalignment, and nonorthogonality are included for both the gyro and accelerometer measurements. Each error is sampled from a zero-mean Gaussian distribution defined by the statistics in Table 2; the bias, scale factor, misalignment, and nonorthogonality sources are modeled to be constant throughout a given trial. Because the primary goal of this work is to compare the propagation architectures, no external measurements are modeled, and only the internal measurements provided by the IMU are simulated. As such, only the prediction stage of the multiplicative extended Kalman filter is employed.

### 4.1. Nominal Simulation

The nominal state propagation considers the case in which the state is propagated at 400 Hz—the frequency at which inertial measurements are sampled. Processing a single measurement is a generally desirable approach for the navigation system, as the algorithmic mechanics tend to be simpler. However, it is almost always desirable to process higher-rate IMU data so that underlying vibration in the vehicle motion can be detected. Unfortunately, the computational resources necessary to process inertial measurements at the rate at which modern inertial sensors are capable of producing them are significant, even when considering state-of-the-art computing systems. Considering this case for the nominal simulation gives a baseline performance for the navigation system’s state and covariance propagation, as it exemplifies the most common method of inertial navigation.

### 4.2. Coning, Sculling, and Scrolling Simulation

The proposed architecture, which employs the CSS algorithms as presented in Section 2 and the error propagation derived within Section 3, can also be used to propagate the mean and covariance of the vehicle state. For simulation, these algorithms operate at a frequency of 10 Hz, while the measurements are simulated at 400 Hz. Therefore, a batch of 40 measurements obtained between $t_{k-1}$ and $t_{k}$ is used to propagate the state and uncertainty of the vehicle through the utilization of second-order CSS correction algorithms. The dynamics outlined in Equation (21) describe the vehicle state evolution.

## 5. Results and Discussion

**Figure 6:**
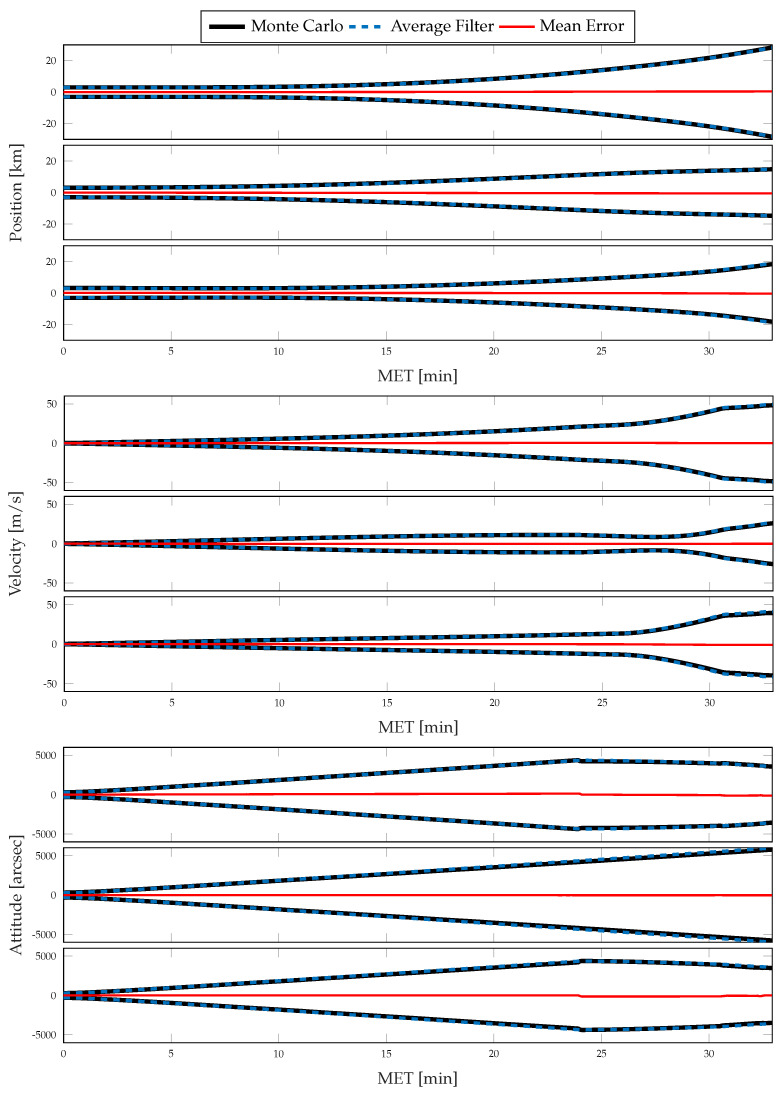
Monte Carlo simulation results from 500 trials using traditional methods of inertial navigation: mean error, averaged filter covariance (3σ), and Monte Carlo sample covariance (3σ) for position (**top**), velocity (**middle**), and attitude (**bottom**).

**Figure 7:**
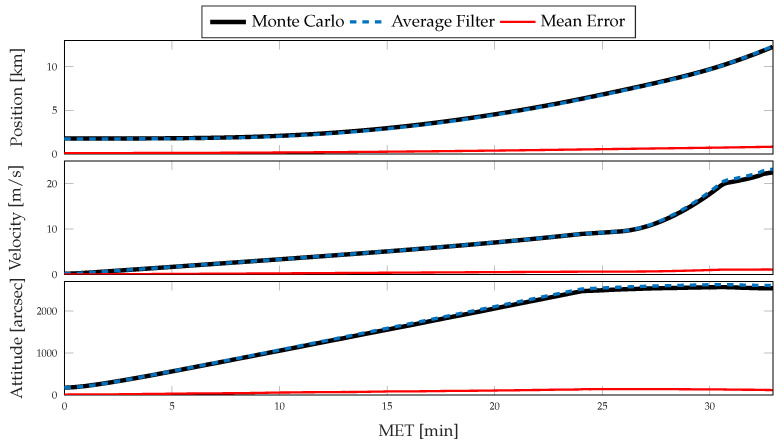
Monte Carlo simulation results from 500 trials using traditional methods of inertial navigation: mean error (RSS), averaged filter covariance (1σ, RSS), and Monte Carlo sample covariance (1σ, RSS) for position (**top**), velocity (**middle**), and attitude (**bottom**).

**Figure 8:**
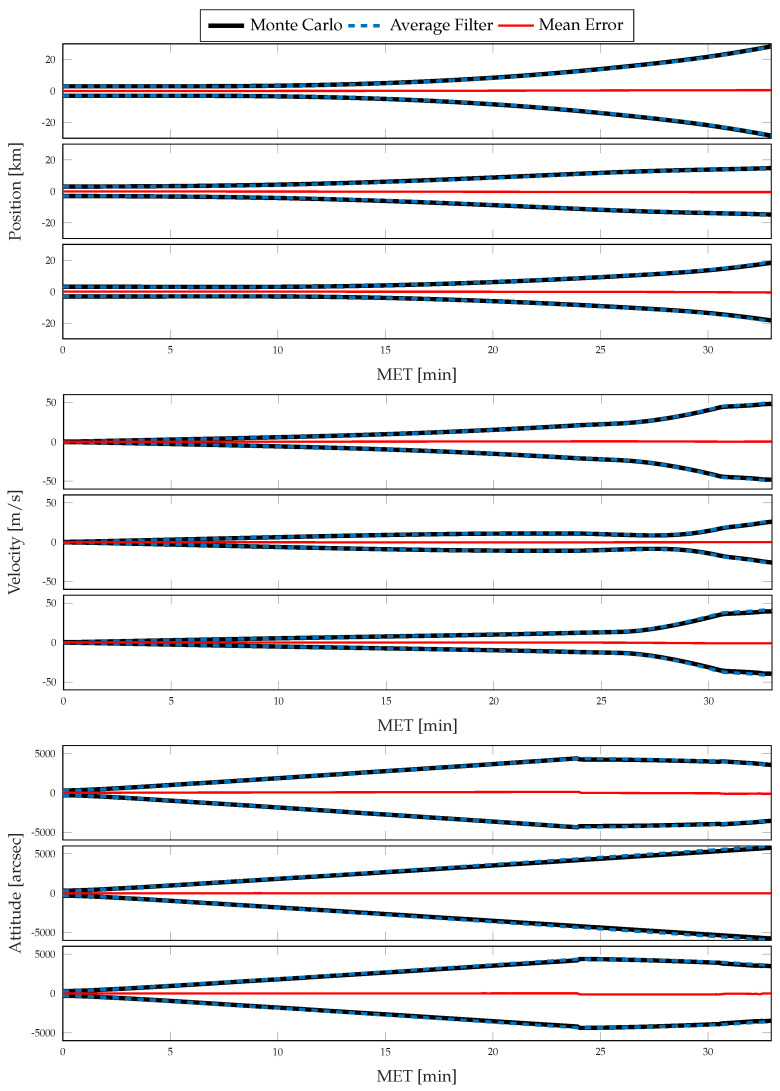
Monte Carlo simulation results from 500 trials using CSS corrections for inertial navigation: mean error, averaged filter covariance (3σ), and Monte Carlo sample covariance (3σ) for position (**top**), velocity (**middle**), and attitude (**bottom**).

**Figure 9:**
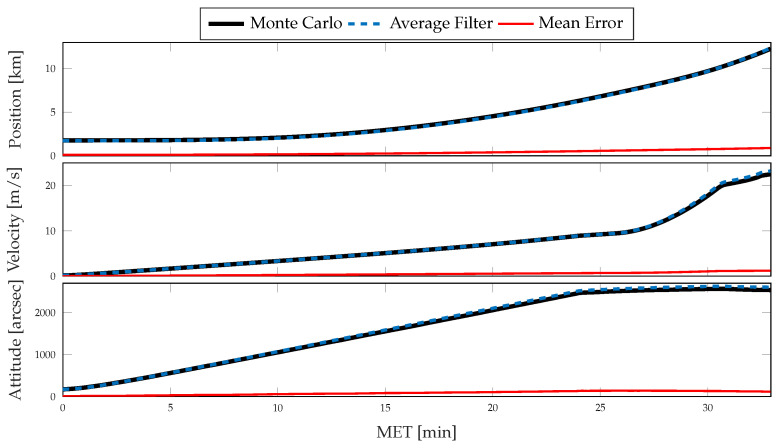
Monte Carlo simulation results from 500 trials using CSS corrections for inertial navigation: mean error (RSS), averaged filter covariance (1σ, RSS), and Monte Carlo sample covariance (1σ, RSS) for position (**top**), velocity (**middle**), and attitude (**bottom**).

For each simulation, the Monte Carlo sample covariance and averaged filter covariance are examined and compared against one another to determine the statistical consistency of the chosen method; i.e., either CSS or the traditional single-measurement dead-reckoning. To better determine how the developed CSS error propagation affects the uncertainty propagation, the same statistics are then compared directly against one another. In Figure 6 and Figure 7, results for the position, velocity, and attitude estimation errors from the nominal simulation are shown. Figure 6 shows the mean estimation error, alongside the averaged filter covariance and Monte Carlo sample covariance 3σ intervals for each component of the position, velocity, and attitude, whereas Figure 7 shows the RSS value for each. Figure 8 and Figure 9 show similar results for the application of the CSS algorithms. From these figures, it is clear that both configurations are consistent with the Monte Carlo statistics, despite the presence of a bias in the mean error. However, by comparing Figure 7 and Figure 9, it is observed that both methods produce similar mean estimation errors with no noticeable difference in the predicted or observed uncertainty intervals. It is worth noting that the mean error not being zero is likely due to under-sampling and continues to reduce with an increasing number of Monte Carlo trials.

**Figure 10:**
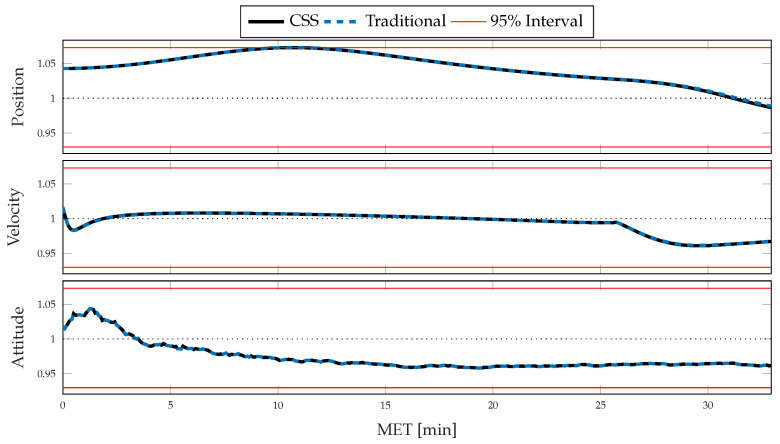
ANEES comparison for Monte Carlo estimation errors generated by CSS and traditional inertial navigation strategies for position (**top**), velocity (**middle**), and attitude (**bottom**).

**Figure 11:**
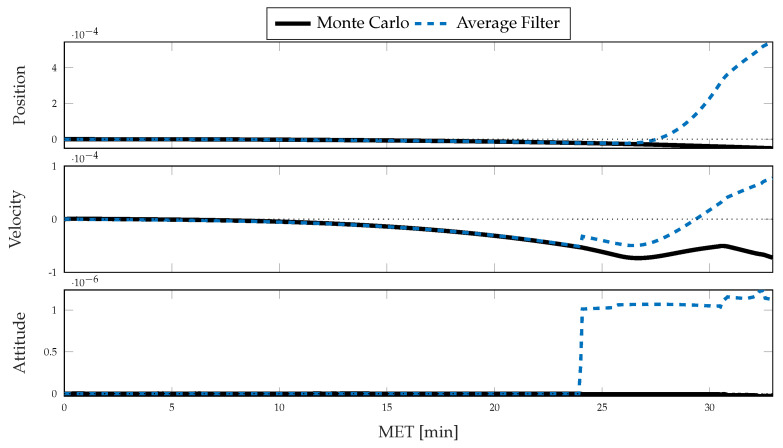
Normalized RSS standard deviation error between CSS and high-frequency traditional dead-reckoning averaged filter and Monte Carlo sample covariances for position (**top**), velocity (**middle**), and attitude (**bottom**).

Within Figure 10 and Figure 11, a direct comparison of each method is made by examining the averaged normalized estimation error squared (ANEES) and a normalized error between the RSS standard deviations. The ANEES, $\bar{\epsilon }$, is calculated by [39,40]

$$\begin{array}{c} \bar{\epsilon }=\frac{1}{nM}\sum_{i=1}^{M}\epsilon_{i}=\frac{1}{nM}\sum_{i=1}^{M}{\left(x_{i}-{\hat{x}}_{i}\right)}^{T}P_{i}^{-1}\left(x_{i}-{\hat{x}}_{i}\right)=\frac{1}{nM}\sum_{i=1}^{M}e_{i}^{T}P_{i}^{-1}e_{i}\;, \end{array}$$

which is the squared Mahalanobis distance for trial *i* of the estimation error, $e_{i}$, with respect to the filter covariance, $P_{i}$, averaged over the number of states, *n*, and the number of Monte Carlo trials, *M*. The ANEES measure is $\chi^{2}$-distributed if the estimation errors are Gaussian-distributed and allows the filter to be rejected as credible at a particular level, α, should a credibility interval be breached for a significant amount of time. The credibility interval is constructed such that $Pr\left(\bar{\epsilon }\in \left[a,b\right]|nM\bar{\epsilon }∼\chi_{nM}^{2}\right)=1-\alpha$ for a<1<b and 0<α≪1, where $\chi_{nM}^{2}$ is a $\chi^{2}$-distribution of nM degrees of freedom [40]. The interval [a,b] contains 95% of the probability mass for the $\chi^{2}$ distribution having a mean of one and nM degrees of freedom when α=0.05. The lower bound *a* separates the lower α/2 of the probability mass, while the upper bound eliminates the upper α/2. If $\bar{\epsilon }=1$, the ANEES is perfectly consistent with the error distribution. It is necessary to note that ANEES is *not* a credibility measure but is useful in recognizing if the filter’s approximation of the uncertainty is representative of the errors or that the filter is consistent [41]. Finally, this measure allows the recognition of estimation performance; the estimator overestimates the estimation error when the ANEES is less than one and underestimates the error when the ANEES is greater than one. While examining the consistency of the diagonal elements of the filter and Monte Carlo sample covariance matrices is useful, ANEES allows the direct comparison of cross-correlation terms in the covariance structure to the estimation errors. Figure 10 shows the ANEES for position, velocity, and attitude for both configurations and allows the assessment that each estimator has approximately the same level of credibility for the estimation of those states. While no significant difference manifests, notice that a slight deviation occurs in the position ANEES around 28 min MET, though no significant difference is noticed in the consistency of the estimators.

Figure 11 compares the RSS standard deviations of the Monte Carlo sample covariance and averaged filter covariance, $\sigma_{x,c}^{rss}$ and $\sigma_{x,t}^{rss}$ respectively, by computing a normalized error, here defined as

$$\begin{array}{c} e_{\sigma ,x}=\frac{\sigma_{x,c}^{rss}-\sigma_{x,t}^{rss}}{\sigma_{x,t}^{rss}}\;, \end{array}$$

between the CSS and traditional configurations for a state *x*, such as position, velocity, or attitude. By this measure, if the value is less than zero, the CSS methods predict or observe a smaller spread in the distribution than the traditional methods and a larger spread if it is greater than zero. Even though the differences are small, several comments can be made when comparing system performance. By recognizing that the ratios of Monte Carlo sample standard deviations are less than zero for the velocity and position distributions and that the mean error is consistent between the two configurations, as recognized by comparing Figure 7 and Figure 9, it is concluded that the application of the CSS algorithms successfully reduces the error present in the system. The same trend is followed by the averaged filter ratio until approximately 24 min MET, when the attitude maneuver occurs. After the attitude maneuver, the vehicle begins the powered-descent phase, and a continued divergence between the ratios is observed. It is therefore clear that the introduction of these algorithms allows for a slightly more conservative representation of the uncertainty during maneuvers, while simultaneously producing a smaller spread in the estimation errors. A caveat to the analysis is that the sample size of 500 Monte Carlo trials is likely somewhat under-sampled, and the significance of the findings made by examining the ratios is difficult to justify as the differences are relatively small.

Comparing the run-time for 500 Monte Carlo trials, a mean run-time of 15.5 min is found for the traditional method and a time of 2.94 min is found for the CSS configuration. The required run-time to process the same measurements and provide the state and covariance at a down-sampled frequency yielded an approximately 81% reduction in computational complexity and scaled as the frequency of covariance propagation is further reduced. Unfortunately, the time savings do not seem to be as large as those shown in [34]. This may be a result of the relatively low number of measurements processed by the CSS algorithms between state predictions; if higher-frequency measurements are available, the reduction is likely to be more significant. Finally, it should be noted that an attempt at streamlining the implementation may also yield an additional run-time reduction.

## 6. Conclusions

Advancements in precision navigation systems are needed to enable the increasingly complex and demanding tasks required for future autonomous vehicles. One method to consider for the improvement of current navigation system capabilities—for the estimation of position, velocity, attitude, and other vehicle-relevant parameters—is the incorporation of previously unmodeled effects, such as the contribution of coning, sculling, and scrolling (CSS) corrections on vehicle position, velocity, and attitude uncertainty within the popular strapdown inertial navigation system (SINS). A method for developing each algorithm’s error propagation has been presented alongside the resulting expressions for a set of CSS algorithms. It is worth noting that the developed error propagation relies upon a linearized error propagation, developed by truncating higher-order terms and assuming that the non-gravitational acceleration and angular velocity vary linearly with time. Thus, this approach is a first-order approximation of the true CSS error propagation—future developments that relax the associated simplifications by incorporating the higher-order effects and allowing more optimized approaches, such as those discussed in [15,25], are of interest for future work.

It is found through Monte Carlo simulation and analysis that the inclusion of an error propagation for the CSS algorithms results in a more conservative filter representation of the predicted position, velocity, and attitude uncertainties following a large attitude maneuver. Additionally, the averaged estimation errors seem consistent with existing methods for the simulated scenario, where both the CSS and traditional dead-reckoning approaches exhibit the same level of statistical consistency. It is also shown that reduced computationally complexity is established by the CSS system when compared to traditional methods of discrete dead-reckoning, giving an 81% reduction in average run-time. If the generation of the error mappings was handled by a navigation pre-processor or within the IMU software, a further reduction of the computational complexity would be seen. Finally, because the developed models for uncertainty propagation allow uncertainty to be handled in a more mathematically consistent manner when using CSS corrections, while simultaneously producing a computationally efficient and statistically conservative navigation filter, it can be concluded that the developed models could be beneficial to future navigation systems.

## Figures and Tables

**Table 1:** Initial uncertainty for each state (per axis, 1σ).

	Uncertainty (1σ)
Position	1000 m
Velocity	0.1 m/s
Attitude	100 arcsec

**Table 2:** Error specifications for the LN-200S IMU (per axis, 1σ).

	Gyroscope	Accelerometer
Frequency	400 Hz	400 Hz
Noise	0.07 ${}^{\circ }/\sqrt{\mathrm{hr}}$	35 $\mu g/\sqrt{\mathrm{hr}}$
Bias	1 ${}^{\circ }/\mathrm{hr}$	300 μg
Scale factor	300 ppm	100 ppm
Misalignment	0.1 mrad	0.1 mrad
Nonorthogonality	0.1 mrad	0.1 mrad

## Data Availability

No new data were created or analyzed in this study. Data sharing is not applicable to this article.

## Appendix A. Incorporating the Strapdown Sensor Model

As discussed in Section 2.5, a number of parameters corrupt the strapdown inertial sensor measurements including bias, scale-factor, axes nonorthogonality, frame misalignment, and white noise. The contribution of each of these common sensor error sources to the measurement error was shown in Equation (29). Using the model in Section 3.2, combined with the state error propagation in Equation (64), the error dynamics for the state can be expressed in terms of the sensor error parameter statistics. Note that throughout this section, it is assumed that the error dynamics for each sensor error parameter are constant, though this assumption is not necessary and is made to somewhat simplify notation.

### Appendix A.1. Attitude

Considering the gyro measurements to be corrupted by bias, scale factor, misalignment, nonorthogonality, and noise error sources, the propagation of these errors into the attitude estimate is given by the combination of Equations (29a) and (64c). Assuming that the parametric errors for the gyroscope have constant dynamics, i.e., that

$$\begin{array}{c} b_{g,k}=b_{g,k-1}\;,\;s_{g,k}=s_{g,k-1}\;,\;m_{g,k}=m_{g,k-1}\;,\;\mathrm{and}\;n_{g,k}=n_{g,k-1}\;, \end{array}$$

the component of Equation (64c) containing the measurement errors is given by

(A1) $$\begin{array}{cc} \sum_{i=1}^{\ell }\left(I_{3\times 3}+\Xi_{con,i}\right)e_{\Delta \theta ,i}= & -L_{s_{g}}e_{s_{g},k}+L_{m_{g}}e_{m_{g},k}-L_{n_{g}}e_{n_{g},k}-L_{b_{g}}e_{b_{g},k}-w_{a,g}\;, \end{array}$$

where the components

(A2a) $$\begin{array}{cc} L_{b_{g}} & =\ell I_{3\times 3}+\sum_{i=1}^{\ell }\Xi_{con,i} \end{array}$$

(A2b) $$\begin{array}{cc} L_{s_{g}} & =\left[\theta_{m,\ell }\setminus \right]+\sum_{i=1}^{\ell }\Xi_{con,i}\left[\Delta \theta_{m,i}\setminus \right] \end{array}$$

(A2c) $$\begin{array}{cc} L_{m_{g}} & =\left[\theta_{m,\ell }\times \right]+\sum_{i=1}^{\ell }\Xi_{con,i}\left[\Delta \theta_{m,i}\times \right] \end{array}$$

(A2d) $$\begin{array}{cc} L_{n_{g}} & =\left[\theta_{m,\ell }*\right]+\sum_{i=1}^{\ell }\Xi_{con,i}\left[\Delta \theta_{m,i}*\right] \end{array}$$

(A2e) $$\begin{array}{cc} w_{a,g} & =\sum_{i=1}^{\ell }\left(I_{3\times 3}+\Xi_{con,i}\right)w_{g,i}\;. \end{array}$$

are defined to simplify the notation and isolate the propagation of the gyro bias, scale factor, misalignment, nonorthogonality, and noise into the attitude estimate, respectively. Note that $\theta_{m,\ell }$ is simply the sum of the measurements; i.e., $\theta_{m,\ell }=\sum_{i=1}^{\ell }\Delta \theta_{m,i}$. The attitude error, including the contribution from each of the gyro error sources, is thus

(A3) $$\begin{array}{cc} e_{a,k} & =T\left(\Delta {\hat{\phi }}_{k}\right)e_{a,k-1}-L_{s_{g}}e_{s_{g},k}+L_{m_{g}}e_{m_{g},k}-L_{n_{g}}e_{n_{g},k}-L_{b_{g}}e_{b_{g},k}-w_{a,g}\;. \end{array}$$

It is important to note that the noise is *not* assumed to be constant over the coning interval and requires special attention for implementation.

### Appendix A.2. Velocity

Given that both the gyros and accelerometers can be corrupted by bias, scale factor, misalignment, nonorthogonality, and noise error sources, the error in the velocity estimate will also be dependent upon the sensor error parameters. Combining Equation (29b) with Equation (64b), the component mapping errors in the accelerometer measurements to the velocity becomes

(A4) $$\begin{array}{cc} \sum_{i=1}^{\ell }{\hat{T}}_{k-1}^{T}\left(N_{\theta }+\Xi_{con,i}\right)e_{\Delta V_{i},k}= & -V_{s_{a}}e_{s_{a},k}+V_{m_{a}}e_{m_{a},k}-V_{n_{a}}e_{n_{a},k}-V_{b_{a}}e_{b_{a},k}-w_{v,a}\;, \end{array}$$

where

(A5a) $$\begin{array}{cc} V_{s_{a}} & ={\hat{T}}_{k-1}^{T}\left\{N_{\theta }\left[v_{m,\ell }\setminus \right]+\sum_{i=1}^{\ell }\Xi_{con,i}\left[\Delta v_{m,i}\setminus \right]\right\} \end{array}$$

(A5b) $$\begin{array}{cc} V_{m_{a}} & ={\hat{T}}_{k-1}^{T}\left\{N_{\theta }\left[v_{m,\ell }\times \right]+\sum_{i=1}^{\ell }\Xi_{con,i}\left[\Delta v_{m,i}\times \right]\right\} \end{array}$$

(A5c) $$\begin{array}{cc} V_{n_{a}} & ={\hat{T}}_{k-1}^{T}\left\{N_{\theta }\left[v_{m,\ell }*\right]+\sum_{i=1}^{\ell }\Xi_{con,i}\left[\Delta v_{m,i}*\right]\right\} \end{array}$$

(A5d) $$\begin{array}{cc} V_{b_{a}} & ={\hat{T}}_{k-1}^{T}\left(\ell N_{\theta }+\sum_{i=1}^{\ell }\Xi_{con,i}\right) \end{array}$$

(A5e) $$\begin{array}{cc} w_{v,a} & =\sum_{i=1}^{\ell }{\hat{T}}_{k-1}^{T}\left(N_{\theta }+\Xi_{con,i}\right)w_{a,i}\;, \end{array}$$

and

(A6) $$\begin{array}{c} N_{\theta }=I_{3\times 3}+\frac{1}{2}\left[{\hat{\theta }}_{\ell }\times \right] \end{array}$$

are defined to simplify notation. Note that this results from assuming that the bias, scale factor, misalignment, and nonorthogonality errors are constant over the major interval, though this assumption can be easily relaxed.

Similarly, by combining Equation (29a) with Equation (64b), defining the mapping of each error in the gyro measurement into the velocity error as

(A7a) $$\begin{array}{cc} v_{b_{g}} & =\sum_{i=1}^{\ell }{\hat{T}}_{k-1}^{T}\Xi_{scul,i}-\frac{1}{2}\left({\hat{T}}_{k-1}^{T}\left[{\hat{v}}_{\ell }\times \right]+\Delta t_{k}{\hat{G}}_{k-1}{\hat{T}}_{k-1}^{T}\left[{\hat{d}}_{k-1}\times \right]L_{s_{b}}\right) \end{array}$$

(A7b) $$\begin{array}{cc} v_{s_{g}} & =\sum_{i=1}^{\ell }{\hat{T}}_{k-1}^{T}\Xi_{scul,i}\left[\Delta \theta_{m,i}\setminus \right]\\ \; & \;\;\;-\frac{1}{2}\left({\hat{T}}_{k-1}^{T}\left[{\hat{v}}_{\ell }\times \right]\left[\theta_{m,\ell }\setminus \right]+\Delta t_{k}{\hat{G}}_{k-1}{\hat{T}}_{k-1}^{T}\left[{\hat{d}}_{k-1}\times \right]L_{s_{g}}\right) \end{array}$$

(A7c) $$\begin{array}{cc} v_{m_{g}} & =\sum_{i=1}^{\ell }{\hat{T}}_{k-1}^{T}\Xi_{scul,i}\left[\Delta \theta_{m,i}\times \right]\\ \; & \;\;\;-\frac{1}{2}\left({\hat{T}}_{k-1}^{T}\left[{\hat{v}}_{\ell }\times \right]\left[\theta_{m,\ell }\times \right]+\Delta t_{k}{\hat{G}}_{k-1}{\hat{T}}_{k-1}^{T}\left[{\hat{d}}_{k-1}\times \right]L_{m_{g}}\right) \end{array}$$

(A7d) $$\begin{array}{cc} v_{n_{g}} & =\sum_{i=1}^{\ell }{\hat{T}}_{k-1}^{T}\Xi_{scul,i}\left[\Delta \theta_{m,i}*\right]\\ \; & \;\;\;-\frac{1}{2}\left({\hat{T}}_{k-1}^{T}\left[{\hat{v}}_{\ell }\times \right]\left[\theta_{m,\ell }*\right]+\Delta t_{k}{\hat{G}}_{k-1}{\hat{T}}_{k-1}^{T}\left[{\hat{d}}_{k-1}\times \right]L_{n_{g}}\right) \end{array}$$

(A7e) $$\begin{array}{cc} w_{v,g} & =\sum_{i=1}^{\ell }{\hat{T}}_{k-1}^{T}\left(\Xi_{scul,i}-\frac{1}{2}\left[{\hat{v}}_{\ell }\times \right]\right)w_{g,i}-\frac{\Delta t_{k}}{2}{\hat{G}}_{k-1}{\hat{T}}_{k-1}^{T}\left[{\hat{d}}_{k-1}\times \right]w_{a,g}\;, \end{array}$$

and incorporating the definitions from Equation (A5), it can be shown that the velocity error propagation is

(A8) $$\begin{array}{cc} e_{v,k}= & \Delta t_{k}\left({\hat{G}}_{k-1}-\frac{1}{2}{\hat{U}}_{k-1}\right)e_{r,k-1}+e_{v,k-1}\\ \; & +\{\frac{\Delta t_{k}}{2}{\hat{g}}_{k-1}{\hat{T}}_{k-1}^{T}\left[\left({\hat{d}}_{k-1}\times \Delta {\hat{\phi }}_{k}\right)\times \right]-{\hat{T}}_{k-1}^{T}\left[\Delta {\hat{v}}_{ng,k}\times \right]\\ \; & \;\;\;\;-\Delta t_{k}\left({\hat{g}}_{k-1}-\frac{1}{2}{\hat{U}}_{k-1}\right){\hat{T}}_{k-1}^{T}\left[{\hat{r}}_{cg/c,k-1}\times \right]\}e_{a,k-1}\\ \; & +\Delta t_{k}\left\{\left({\hat{g}}_{k-1}-\frac{1}{2}{\hat{U}}_{k-1}\right){\hat{T}}_{k-1}^{T}+\frac{1}{2}{\hat{g}}_{k-1}{\hat{T}}_{k-1}^{T}\left[\Delta {\hat{\phi }}_{k}\times \right]\right\}e_{d,k-1}\\ \; & -v_{s_{a}}e_{s_{a},k}+v_{m_{a}}e_{m_{a},k}-v_{n_{a}}e_{n_{a},k}-v_{b_{a}}e_{b_{a},k}-w_{v,a}\\ \; & -v_{s_{g}}e_{s_{g},k}+v_{m_{g}}e_{m_{g},k}-v_{n_{g}}e_{n_{g},k}-v_{b_{g}}e_{b_{g},k}-w_{v,g}\;. \end{array}$$

Therefore, the error propagation in Equation (A8) describes the error propagation for the velocity estimate given that the navigation system is dependent upon strapdown inertial sensors and uses a coning and sculling algorithm, where the sensor errors are assumed constant over the timestep.

### Appendix A.3. Position

With the definition of the velocity error dynamics written in terms of the strapdown sensor errors in Equation (A8), their propagation into the position error follows similarly. The mappings defining the propagation of gyro measurement errors into the position estimate are thus defined as

(A9a) $$\begin{array}{cc} r_{b_{g}} & =\sum_{i=1}^{\ell }{\hat{T}}_{k-1}^{T}X_{\Delta \theta ,i}-\frac{\Delta t_{k}^{2}}{6}{\hat{G}}_{k-1}{\hat{T}}_{k-1}^{T}\left[{\hat{d}}_{k-1}\times \right]L_{b_{g}} \end{array}$$

(A9b) $$\begin{array}{cc} r_{s_{g}} & =\sum_{i=1}^{\ell }{\hat{T}}_{k-1}^{T}X_{\Delta \theta ,i}\left[\Delta v_{m,i}\setminus \right]-\frac{\Delta t_{k}^{2}}{6}{\hat{G}}_{k-1}{\hat{T}}_{k-1}^{T}\left[{\hat{d}}_{k-1}\times \right]L_{s_{g}} \end{array}$$

(A9c) $$\begin{array}{cc} r_{m_{g}} & =\sum_{i=1}^{\ell }{\hat{T}}_{k-1}^{T}X_{\Delta \theta ,i}\left[\Delta v_{m,i}\times \right]-\frac{\Delta t_{k}^{2}}{6}{\hat{G}}_{k-1}{\hat{T}}_{k-1}^{T}\left[{\hat{d}}_{k-1}\times \right]L_{m_{g}} \end{array}$$

(A9d) $$\begin{array}{cc} r_{n_{g}} & =\sum_{i=1}^{\ell }{\hat{T}}_{k-1}^{T}X_{\Delta \theta ,i}\left[\Delta v_{m,i}*\right]-\frac{\Delta t_{k}^{2}}{6}{\hat{G}}_{k-1}{\hat{T}}_{k-1}^{T}\left[{\hat{d}}_{k-1}\times \right]L_{n_{g}} \end{array}$$

(A9e) $$\begin{array}{cc} w_{r,g} & =\sum_{i=1}^{\ell }{\hat{T}}_{k-1}^{T}X_{\Delta \theta ,i}w_{a,i}-\frac{\Delta t_{k}^{2}}{6}{\hat{G}}_{k-1}{\hat{T}}_{k-1}^{T}\left[{\hat{d}}_{k-1}\times \right]w_{a,g}\;. \end{array}$$

while the mapping for the errors in the accelerometer measurements are defined as

(A10a) $$\begin{array}{cc} r_{b_{a}} & =\sum_{i=1}^{\ell }{\hat{T}}_{k-1}^{T}X_{\Delta v,i} \end{array}$$

(A10b) $$\begin{array}{cc} r_{s_{a}} & =\sum_{i=1}^{\ell }{\hat{T}}_{k-1}^{T}X_{\Delta v,i}\left[\Delta \theta_{m,i}\setminus \right] \end{array}$$

(A10c) $$\begin{array}{cc} r_{m_{a}} & =\sum_{i=1}^{\ell }{\hat{T}}_{k-1}^{T}X_{\Delta v,i}\left[\Delta \theta_{m,i}\times \right] \end{array}$$

(A10d) $$\begin{array}{cc} r_{n_{a}} & =\sum_{i=1}^{\ell }{\hat{T}}_{k-1}^{T}X_{\Delta v,i}\left[\Delta \theta_{m,i}*\right] \end{array}$$

(A10e) $$\begin{array}{cc} w_{r,a} & =\sum_{i=1}^{\ell }{\hat{T}}_{k-1}^{T}X_{\Delta v,i}w_{a,i}\;. \end{array}$$

With the mappings defined in Equations (A9) and (A10), the error propagation for the position becomes

(A11) $$\begin{array}{cc} e_{r,k}= & \left\{I_{3\times 3}+\frac{\Delta t_{k}^{2}}{2}\left({\hat{G}}_{k-1}-\frac{1}{3}{\hat{U}}_{k-1}\right)\right\}e_{r,k-1}+\Delta t_{k}e_{v,k-1}\\ \; & +\{\frac{\Delta t_{k}^{2}}{6}{\hat{T}}_{k-1}^{T}\left[\left({\hat{d}}_{k-1}\times \Delta {\hat{\phi }}_{k}\right)\times \right]-{\hat{T}}_{k-1}^{T}\left[\Delta {\hat{r}}_{ng,k}\times \right]\\ \; & \;\;\;\;\;-\frac{\Delta t_{k}^{2}}{2}\left({\hat{G}}_{k-1}-\frac{1}{3}{\hat{U}}_{k-1}\right){\hat{T}}_{k-1}^{T}\left[{\hat{d}}_{k-1}\times \right]\}e_{a,k-1}\\ \; & +\frac{\Delta t_{k}^{2}}{2}\left\{\left({\hat{G}}_{k-1}-\frac{1}{3}{\hat{U}}_{k-1}\right){\hat{T}}_{k-1}^{T}+\frac{1}{3}{\hat{G}}_{k-1}{\hat{T}}_{k-1}^{T}\left[\Delta {\hat{\phi }}_{k}\times \right]\right\}e_{d,k-1}\\ \; & -r_{s_{a}}e_{s_{a},k}+r_{m_{a}}e_{m_{a},k}-r_{n_{a}}e_{n_{a},k}-r_{b_{a}}e_{b_{a},k}-w_{r,a}\\ \; & -r_{s_{g}}e_{s_{g},k}+r_{m_{g}}e_{m_{g},k}-r_{n_{g}}e_{n_{g},k}-r_{b_{g}}e_{b_{g},k}-w_{r,g}\;. \end{array}$$

Equation (A11) the error dynamics for the position estimate, influenced by accelerometer and gyro bias, scale factor, misalignment, nonorthogonality, and white noise—the bias, scale factor, misalignment, and nonorthogonality sensor error are assumed to be constant from $t_{k-1}$ to $t_{k}$. The noise, however, is not assumed constant over the interval and thus provides a separate contribution from each measurement, requiring the definition of $w_{r,g}$ and $w_{r,a}$.
